# The nematode (*Ascaris suum*) intestine is a location of synergistic anthelmintic effects of Cry5B and levamisole

**DOI:** 10.1371/journal.ppat.1011835

**Published:** 2024-05-17

**Authors:** Paul D. E. Williams, Matthew T. Brewer, Raffi V. Aroian, Alan P. Robertson, Richard J. Martin

**Affiliations:** 1 Department of Biomedical Sciences, Iowa State University, Ames, Iowa, United States of America; 2 Department of Veterinary Pathology, Iowa State University, Ames, Iowa, United States of America; 3 Program in Molecular Medicine, University of Massachusetts Chan Medical School, Worcester, Massachusetts, United States of America; Rush University Medical Center, UNITED STATES

## Abstract

A novel group of biocidal compounds are the Crystal 3D (Cry) and Cytolytic (Cyt) proteins produced by *Bacillus thuringiensis* (Bt). Some Bt Cry proteins have a selective nematocidal activity, with Cry5B being the most studied. Cry5B kills nematode parasites by binding selectively to membrane glycosphingolipids, then forming pores in the cell membranes of the intestine leading to damage. Cry5B selectively targets multiple species of nematodes from different clades and has no effect against mammalian hosts. Levamisole is a cholinergic anthelmintic that acts by selectively opening L-subtype nicotinic acetylcholine receptor ion-channels (L-AChRs) that have been found on muscles of nematodes. A synergistic nematocidal interaction between levamisole and Cry5B at the whole-worm level has been described previously, but the location, mechanism and time-course of this synergism is not known. In this study we follow the timeline of the effects of levamisole and Cry5B on the Ca^2+^ levels in enterocyte cells in the intestine of *Ascaris suum* using fluorescence imaging. The peak Ca^2+^ responses to levamisole were observed after approximately 10 minutes while the peak responses to activated Cry5B were observed after approximately 80 minutes. When levamisole and Cry5B were applied simultaneously, we observed that the responses to Cry5B were bigger and occurred sooner than when it was applied by itself. It is proposed that the synergism is due to the cytoplasmic Ca^2+^ overload that is induced by the combination of levamisole opening Ca^2+^ permeable L-subtype nAChRs and the Ca^2+^ permeable Cry5B toxin pores produced in the enterocyte plasma membranes. The effect of levamisole potentiates and speeds the actions of Cry5B that gives rise to bigger Ca^2+^ overloads that accelerates cell-death of the enterocytes.

## Author summary

The neglected tropical diseases are a diverse set of infectious diseases which are common in low-income populations of Asia, Africa and the Americas. They include soil-transmitted helminth (STH) infections produced by the parasitic nematode *Ascaris lumbricoides* (= *Ascaris suum*). There are no effective vaccines, so mass drug administration (***MDA***) to control and prevent infection is the only practical option. With the limited number of anthelmintic drugs available for treatment, there is an increasing concern about the development of resistance. The use of combinations of anthelmintics, particularly if they are additive, is important for the delay of the onset of resistance. Here we describe synergistic interactions of levamisole and Cry5B mediated by Ca^2+^ in *A*. *suum* intestine enterocytes. We have been able to make these new observations because of our access to *A*. *suum* parasites and development of our techniques for studying Ca^2+^ signaling in the intestine of this nematode parasite, a site of the interaction of these two anthelmintic drugs. Our study provides new insights for development of effective STH combination therapies of anthelmintic drugs for slowing development of resistance with continued mass drug administration and understanding the nematode parasite intestine as a major target for combinations of anthelmintic drugs.

## Introduction

Infections by soil-transmitted helminths (STHs), including: the roundworm *Ascaris lumbricoides*, the whipworm *Trichuris trichiura*, and hookworms (*Ancylostoma ceylanicum* and *Necator americanus*) are major medical and public health concerns in many developing countries. It is estimated that 807 million to 1.2 billion people are infected with the large roundworm *A*. *lumbricoides* [1]. Although rarely fatal, parasitic infections have a detrimental effect on morbidity, currently reducing worker productivity by 3.5 million **D**isability **A**djusted **L**ife **Y**ears (DALYs) per year [2] and have a severe impact on children due to stunting of physical growth, cognitive impairment, malnutrition and anemia [1]. In livestock, infestations lead to reduced food yields, impacting of economic returns and the exacerbation of poverty [3].

Current treatments rely on the use of chemotherapeutics, usually using one of the three major classes of anthelmintics: benzimidazoles (*e*.*g*., albendazole/mebendazole), macrocyclic lactones (*e*.*g*., ivermectin), or nicotinic cholinergics (*e*.*g*., levamisole) because there are no effective vaccines. Resistance to these anthelmintics is frequently observed in domestic animals [4] and there is a real concern that resistance will present in parasites of humans [5,6]. With the limited number of anthelmintic drugs and the concerns about the development of resistance, it is important to: 1) seek novel classes of anthelmintics that have different modes of action; and 2) to identify mechanism-based rational anthelmintic combinations that are more potent and effective.

*Bacillus thuringiensis* (Bt) three-domain crystal (Cry) proteins are a potential novel class of anthelmintic. It is known that Bt Cry proteins form pores in the intestine of insect pests and are used successfully for insect control. Once ingested by insects, these toxins are solubilized in their midgut, and activated by proteases to yield the active toxin. The toxin then binds to various membrane receptors including glycopeptides [7,8]. The toxin is then transported into the plasma membrane of intestinal cells of the insect where it forms oligomeric pores that cause ion leakage and cell lysis [7]. A major advantage of the Cry proteins is that they are very specific, targeting only invertebrates and are therefore safe for use in vertebrates even at high concentrations as vertebrates lack these glycoprotein receptors [9–11]. The potency and biosafety of the Bt Cry protein toxins have resulted in them being used widely for insect pest control in transgenic food crops.

A selection of Bt Cry proteins has been identified as being effective at targeting free-living and parasitic nematodes [12–17]. Cry5B is the most studied of these nematode specific Cry proteins. It acts on the intestine of the nematode as a pore forming toxin by binding to glycosphingolipid receptors created by BRE-5 [18–20] and cadherin CDH-8 receptors [21]. After binding to the receptors, the toxin oligomerizes to form a pore that disrupts the integrity of the intestine. Combinations of the anthelmintic levamisole and Cry5B have synergistic lethal effects on *C*. *elegans* and enhanced clearing of nematode parasites when compared to either of the compounds used alone [22,23]. Although interactions between levamisole and Cry5B have been described at the whole worm level, the location, mechanism and time-course of the interactions require further study.

The intestine of nematode parasites serves as a barrier that limits access of ingested xenobiotics in the intestine to adjacent muscles and nerves. The barrier is due to the presence of the tight junctions between the enterocytes, the ability of the enterocytes to metabolize xenobiotic compounds, and the excretion by transporters of xenobiotics back into the intestine. The synergistic interactions on the whole nematode of Cry5B and levamisole after ingestion, might be explained by: a direct synergist interaction of Cry5B and levamisole on the enterocytes of the intestine; or Cry5B damaging the barrier properties of the intestine that then allows increased access of levamisole in the intestine to target receptors of the adjacent muscle and nerves.

Here we examine effects on the Ca^2+^ signals of the isolated intestine and show that Cry5B and levamisole together have a direct synergistic effect on the cells of the intestine of the parasitic nematode *Ascaris suum*, which appears genetically and phenotypically to be the same species as *A*. *lumbricoides* of humans [24]. We observed over 6 hours that Cry5B has concentration-dependent and time-dependent effects on the amplitude of the Ca^2+^ signals of the intestine that are related to its histological damage. We explored the time course of the effects of combining levamisole and Cry5B on the Ca^2+^ signals and histology of the intestine. Combination of levamisole and Cry5B produced quicker and bigger effects on Ca^2+^ signals and histology than Cry5B alone. We identified the intestine as a shared site of action for the cholinergic anthelmintic levamisole and activated Cry5B. Our observations show that it is interactions on the enterocytes that produce a synergistic effect and that it is not only a synergism being produced by increased access of ingested levamisole to muscle and nerves following damage to the intestine.

## Materials and methods

### Collection and maintenance of *A. suum* worms

Adult female *A*. *suum* worms were collected from the JBS Swift and Co. pork processing plant at Marshalltown, Iowa. Worms were maintained in *Ascaris* Ringers Solution (ARS: 13 mM NaCl, 9 mM CaCl_2_, 7 mM MgCl_2_, 12 mM C_4_H_11_NO_3_/Tris, 99 mM NaC_2_H_3_O_2_, 19 mM KCl and 5 mM glucose pH 7.8) at 32°C for 24 hours to allow for acclimatization before use in experiments. The solution was changed daily, and worms were used within three days for experiments. All the worms were examined at the start of each day and removed if they were damaged or immotile.

### Dryad DOI

https://doi.org/10.5061/dryad.b5mkkwhkk

### *A. suum* cDNA synthesis and RT-PCR detection of Cry5B target mRNA

Dissection of body wall and intestinal tissue was conducted on adult *A*. *suum* females as previously described [25,26]. The body wall and intestinal tissue were homogenized separately in 1 ml of Trizol reagent using a mortar and pestle, followed by total RNA extraction according to the Trizol Reagent protocol (Life Technologies, USA). One microgram (1 μg) of total RNA from each tissue was used to generate cDNA by reverse transcription (RT) using SuperScript IV Master Mix (Life Technologies, USA) following the manufacturer’s protocol. PCR was conducted to detect the presence of *Asu-bre-5* and *Asu-cdh-8* using primers targeting coding regions of each gene (S1 Table). *Asu-gapdh* was used as a reference gene. The negative controls used no cDNA template. The PCR conditions were an initial denaturation for 2 min at 98°C, followed by 35 cycles of 98°C for 30 sec, 58°C for 35 sec, 72°C for 45 sec, and a final extension at 72°C for 10 min using G2 Hot Start Green Master Mix (Promega, USA). The PCR products of each gene were then separated on 2% agarose gels containing Safe DNA Gel Stain (ThermoFisher Scientific), at 100V, followed by visualization under UV light to confirm the presence of the genes. All photographs were acquired using software (Analytik Jena) with an exposure setting of 3 seconds per 1 frame. Original gel pictures are presented in S1 Fig.

### Analysis of mRNA levels by Quantitative Real-time PCR

We quantified mRNA transcript levels of *Asu-bre-5* and *Asu-cdh-8* in the intestine and body wall of adult female *A*. *suum*. Amplicons ranging from 150 to 200 bp were generated by qPCR from each cDNA sample in triplicate. *Asu-gapdh* was used as the reference gene (for qPCR primers see S2 Table). The quantitative PCR reaction mixture consisted of 1 μl of cDNA template, 1 μl of the forward and reverse primer, and 10 μl of Green Master Mix (Applied Biosystems, ThermoFisher, USA), with the final volume made up to 20 μl with nuclease-free water. The thermocycler conditions included an initial denaturation for 10 seconds at 98°C, 40 cycles of 98°C for 15 seconds and 58°C for 30 seconds followed by a final melting curve step. Cycling was performed using a 3 96 well 0.1 mL Block Real time PCR Detection system (ThermoFisher, USA), and transcript quantities were derived by the system software, using the generated standard curves. mRNA expression levels for each gene (*Asu-bre-5* and *Asu-cdh-8*) were estimated relative to the reference gene (*Asu-gapdh*) using the Pfaffl Method [27]. The qPCR experiments were repeated 3 times for each gene (all subunit mRNA quantifications were performed in triplicate for each worm’s body wall sample and intestinal tissue sample: 15 individual female worms providing a body wall and intestinal sample, each from a mean of three technical replicates). The sequence data analyzed for this study are available in Wormbase Parasite, The European Nucleotide Archive (ENA) and UNIPROT repositories, https://parasite.wormbase.org/index.html, www.elixir-europe.org/platforms/data/core-data-resources, www.uniprot.org. Links to the datasets are presented in S3 Table.

### Preparation and loading Fluo-3AM

**Fig 1:**
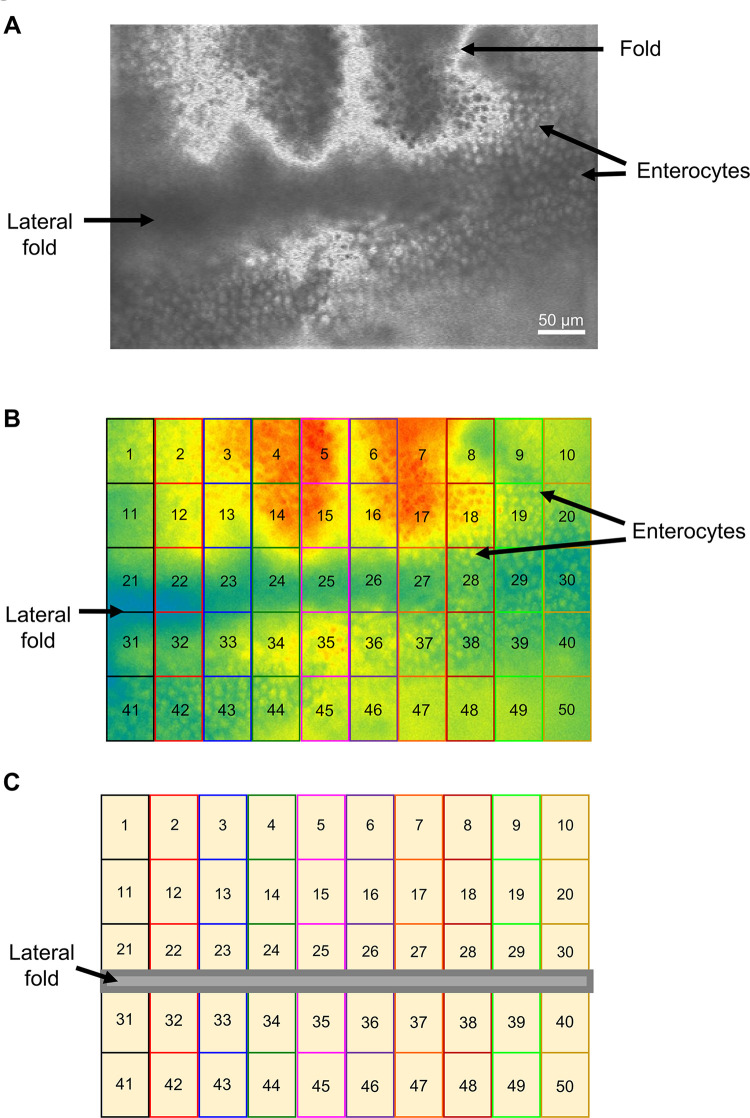
Illustration of intestinal regions. A) Photograph of intestinal preparation under white light. Key structures are highlighted. B) Photograph of the same intestinal preparation shown in (A) under blue light after Fluo-3AM treatment and Ca^2+^ loading, highlighted with the 50 regions used to record Ca^2+^ signals. C) Cartoon illustration of (B) highlighting the 50 regions used to record from a signal intestinal piece. The lateral fold line is included.

Intestinal tissues were loaded with Fluo-3AM as previously described [26]. Briefly, a 2 cm section of the intestine was removed from the body piece using fine forceps and cut open. The intestinal flap was placed in a Warner RC26G recording chamber (Warner Instruments, Hamden, CT) and immobilized using a 26 x 1mm x 1.5mm grid slice anchor (Warner Instruments, Hamden, CT), bathed in *Ascaris* Perienteric Fluid APF (23 mM NaCl, 110 mM NaAc, 24 mM KCl, 1 mM CaCl_2_, 5 mM MgCl_2_, 5 mM HEPES, 11 mM D-glucose) (Fig 1A). The chamber temperature was maintained at 34–36°C using a Dual Automatic Temperature Controller and inline heater (Warner Instruments, Hamden, CT). Fluo-3AM loading was achieved by incubating the intestine in APF solution with no added CaCl_2_ ([Ca^2+^] <100 μM) containing 5 μM Fluo-3AM and 10% Pluronic F-127 (10% v/v) for one hour. After incubation, the Fluo-3AM solution was discarded, and the sample was incubated in APF that contained 1 mM CaCl_2_ for an additional 15 minutes at 34–36°C to promote Ca^2+^ loading (Fig 1B). All incubations were done in the absence of light to prevent degradation of the fluorescent dye. At the start of all experiments, intestinal preparations were left under blue light for a minimum of 3 minutes to promote settling and equilibration of the fluorescent signal and monitor for any spontaneous Ca^2+^ signals before application of any compound. For the recordings, the APF was removed using an exhaust line and the sample was exposed to either: 1) fresh 1 mM CaCl_2_ APF for 6 hours; 2) fresh 1 mM CaCl_2_ APF + 100 μg/ml Cry5B for 6 hours; 3) fresh 1 mM CaCl_2_ APF + 10 μg/ml Cry5B for 6 hours; 4) fresh 1 mM CaCl_2_ APF + 100 μg/ml Cry5B and 100 mM galactose for 6 hours; 5) fresh 1 mM CaCl_2_ APF + 10 μg/ml Cry5B for 2 hours; 6) fresh 1 mM CaCl_2_ APF + 30 μM levamisole for 2 hours; 7) fresh 1 mM CaCl_2_ APF + 30 μM levamisole + 10 μg/ml Cry5B for 2 hours. For the triple combination experiments with mecamylamine, levamisole and Cry5B, samples were pre-incubated in APF containing 10 μM mecamylamine in fresh 1 mM CaCl_2_ APF for 10 minutes after Fluo-3AM incubation. This 10 μM mecamylamine pre-incubation solution was removed using the exhaust line and replaced with fresh 1 mM CaCl_2_ APF + 10 μM mecamylamine + 30 μM levamisole + 10 μg/ml Cry5B for 2 hours. The solution level of the chamber was constantly maintained throughout the recordings. The temperature of the chamber was maintained between 34–36°C for the entire recording, any samples where the temperature deviated from this range were discarded. All solutions were delivered to the chamber using transfer pipettes away from the intestinal preparation.

### Measurement of Ca^2+^ fluorescence

All recordings were performed with a Nikon Eclipse TE300 microscope (20X/0.45 Nikon PlanFluor objective), fitted with a Photometrics Retiga R1 camera (Photometrics, Surrey, BC, Canada). Light control was achieved using a Lambda 10–2 two filter wheel system with a shutter controller (Lambda Instruments, Switzerland). Filter wheel one was set on a green filter (510–560 nM bandpass, Nikon USA) between the microscope and camera. Filter wheel two set on the blue filter (460–500 nM, bandpass, Nikon, USA) between a Lambda LS Xenon bulb light box which delivered light via a fiber optic cable to the microscope (Lambda Instruments, Switzerland). The blue light emission was controlled using a shutter. All Ca^2+^ signal recordings were acquired and analyzed using MetaFluor 7.10.2 (MDS Analytical Technologies, Sunnyvale, CA). Exposure times were 150 ms with 2x binning. Maximal Ca^2+^ signal amplitudes (ΔF/F_0_%) for all stimuli applied were calculated using the equation F-F0/F0 where F is the fluorescent value and F0 is the baseline fluorescent value, which was determined as the lowest value before the largest rise in fluorescence for all recordings analyzed. Representative traces were generated using the same formula, with F0 being the value before a detectable increase in the fluorescence. For the 1 mM CaCl_2_ bath solution control experiments F0 was determined to be the value before the largest rise in Ca^2+^. For the control CaCl_2_ responses F0 was determined as the value before stimulus application. Time-to-peak was calculated by normalizing the trace during stimulus exposure, with the lowest fluorescence value being represented by 0% and the highest being 100%. The time-to-peak was determined by subtracting the time when the stimulus was applied from the time the signal reached 100%.

### Regions used for measurements of the Ca^2+^ signals and % response area

Ca^2+^ signals from each intestine were collected from 50 squares each 50 μm x 50 μm from rectangular 125,000 μm^2^ areas of the intestine that included 800–1000 enterocytes (Fig 1B and 1C). The relative fluorescence amplitude was determined and followed over time for each of the individual 50 square regions. Long-term control preparations were not exposed to any test agents and were incubated in 1 mM CaCl_2_ APF and followed over 6 hours. A 10 mM CaCl_2_ test pulse was added to the chamber as a test of viability of the preparation. An increase >10% in relative Fluo-3 fluorescence to the 10 mM CaCl_2_ pulse was taken as an indication of the positive health of the preparation: while preparations were rejected if the responses to 10 mM CaCl_2_ were <10% as not being viable. For the 6-hour incubations, intestines were exposed to either 10 μg/ml Cry5B, 100 μg/ml Cry5B, or 100 μg/ml Cry5B and 100 mM galactose. For the 2-hour recordings preparations were exposed to either 10 μg/ml Cry5B, 30 μM levamisole as the sole active agent or a combination of 10 μg/ml Cry5B and 30 μM levamisole. For the triple combination of mecamylamine, levamisole and Cry5B, samples were incubated in APF containing 10 μM mecamylamine for 10 minutes after Fluo-3AM incubation, to promote inhibition. After 3 minutes of recording, the mecamylamine APF solution was discarded and replaced with APF containing 10 μM mecamylamine, 30 μM levamisole and 10 μg/ml Cry5B and samples were recorded for 2 hours. Any of the 50 μm x 50 μm regions whose Ca^2+^ amplitude responses to the anthelmintic stimulus that was smaller than the average amplitude of the spontaneous Ca^2+^ signals (2.4% ± 0.1%) were discarded. The reason for their rejection was that we could not rule out the possibility that these signals were, themselves, spontaneous rather than produced by the anthelmintic stimulus. The datasets analyzed for the figures are available at Dryad [28].

### Histology

Sections of *A*. *suum* body were cut along the lateral line and opened, exposing the intestine. For 6-hour comparisons, sections were incubated in either: APF for 2 or 6 hours; APF containing 100 μg/ml Cry5B for 2 or 6 hours; APF containing 10 μg/ml Cry5B for 2 or 6 hours; or APF containing 100 μg/ml Cry5B and 100 mM galactose for 2 or 6 hours. For the two-hour incubations samples were incubated in: APF containing 10 μg/ml Cry5B for 2 hours, APF containing 30 μM levamisole for 2 hours; or APF containing 10 μg/ml Cry5B and 30 μM levamisole for 2 hours.

Following treatment, the segments of adult *A*. *suum* worms were preserved in 10% buffered neutral formalin. Preserved specimens were placed in histology cassettes for paraffin embedding and sectioning. Hematoxylin and eosin staining was performed on 5 μm slides [29]. Sections were viewed and images were captured on an Olympus BX43 microscope fitted with an Olympus DP23 camera (Olympus, Tokyo, Japan). Image captures were achieved using Olympus DP controller software (Olympus, Tokyo, Japan) under 20x magnification and exposure settings of 1/350 seconds per frame.

### Preparation of Cry5B

Cry5B was supplied by Raffi Aroian in both active and inactive form. Inactive Cry5B was activated using elastase at a Cry5B to elastase mass ratio of 200:1, overnight at room temperature [30]. Both active and inactive Cry5B were stored at -80°C suspended in 20 mM HEPES (pH 8.0). Cry5B solutions were made fresh for each experiment.

### Statistical analysis

Statistical analysis of all data was made using GraphPad Prism 9.0 (Graphpad Software, Inc., La Jolla, CA, USA). We used a minimum of 5 individual intestines from 5 individual female worms for all experiments and treatments, with a total of 50 regions for each intestine. The total number of female worm intestinal preparations, the total number of regions showing responses, the concentrations, and durations of applications are provided in the figure legends of the figures. Analysis of Ca^2+^ amplitudes and time-to-peak were made using either unpaired for separate preparations or paired when the responses in the same preparation were being followed using student *t*-tests *P <* 0.05. The *t*-test (paired or unpaired) used is stated in the figure legends.

### Chemicals

Source of chemicals: Cry5B was provided by Raffi Aroian. Galactose and levamisole were procured from Sigma Aldrich. Fisher Scientific supplied all other chemicals.

## Results

### Cry5B targets are present in *Ascaris suum* intestine

The *Bacillus thuringiensis* (Bt) bacteria produce different pore forming toxins referred to as Crystal 3D or Cry proteins. Cry5B is a Cry protein that is nematocidal and is understood to have a similar mode of action as the insecticidal Cry proteins. Previous studies have described receptors for Cry5B that are: 1) glycosphingolipids that are produced by the acetylglucosaminyltransferase activity of BRE-5 [18–20] and; 2) the cadherin CDH-8 which are responsible for oligomerization of Cry5B [21]. Loss of both genes results in a level of resistance to Cry5B in *C*. *elegans*.

**Fig 2:**
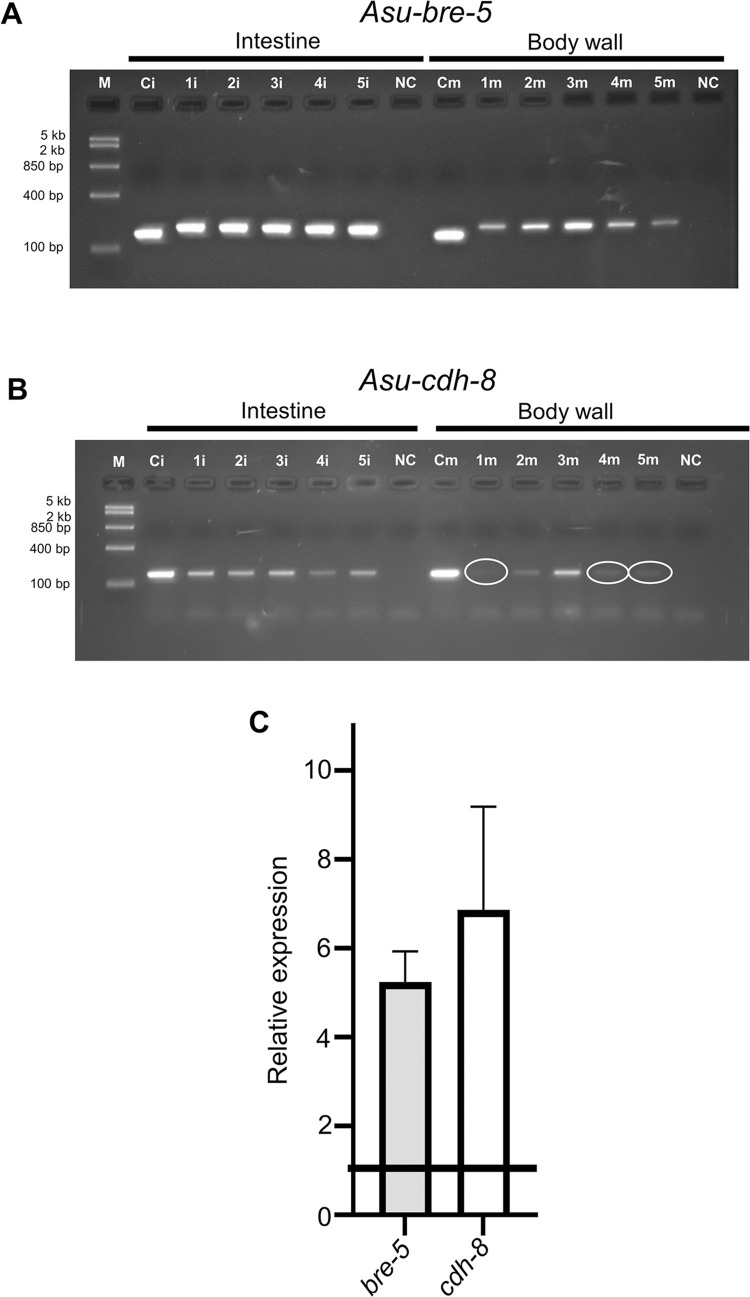
Cry5B message targets are present in *A*. *suum* intestine. RT-PCR analysis of intestine (1i, 2i, 3i, 4i, 5i) and body wall (1m, 2m, 3m, 4m, 5m) of five separate female *A*. *suum* worms. Each lane represents the intestine or body wall of an individual worm. *Asu-gapdh* from the intestine (Ci) and body wall (Cm) was used as a positive control. N.C. = no template negative control. M = Fast Ruler Middle Range DNA Ladder (TermoFisher Scientific). A) *Asu-bre-5* and B) *Asu-cdh-8*. Note reduced intensity of bands highlighted by white circles in the body wall samples. All images are cropped. Images were taken under UV light with an exposure setting of 3 s per 1 frame. Original gel images are presented in S1 Fig. C) Differential expression of Cry5B targets between intestine and body wall. Bar chart (expressed as mean ±SEM) demonstrating transcript level analysis from intestinal samples for *bre-5* (grey bar) (5.24 ± 0.68) and *cdh-8* (white bar) (6.86 ± 2.32) when compared to paired body wall samples. n = 15 individual worms from 3 separate groups of *A*. *suum*, collected at different times.

To determine if BRE-5 and CDH-8 could be present in *A*. *suum*, we blasted the *C*. *elegans* orthologues against the *A*. *suum* genome and identified the predicted genes, *Asu-bre-5* and *Asu-cdh-8* (S3 Table). To determine if message for these genes is expressed in the intestine and/or body wall, we generated cDNA from RNA pools from the intestine and body wall and screened using primers targeting *Asu-bre-5* and *Asu-cdh-8*, Fig 2A and 2B (S1 Table). Fig 2 shows that *Asu-bre-5*, (Fig 2A), and *Asu-cdh-8*, (Fig 2B), were present in both the intestine and body wall in all pools tested. The intensity of the bands in the intestine was higher than the body wall for both *bre-5* and *cdh-8*.

To determine if expression of *bre-5* and *cdh-8* were higher in the intestinal tissue, we performed qPCR comparing the relative expression level between the body wall and the intestine. Fig 2C illustrates the relative expression levels of *Asu-bre-5* and *Asu-cdh-8* which were higher in the intestine than the body wall (5.2-fold for *bre-5* and 6.9-fold for *cdh-8*). These observations suggest that the Cry5B targets are conserved in *A*. *suum* and that they have a higher level of transcript expression in the intestine. The presence of *Asu-bre-5* and *Asu-cdh-8* message in the body wall of the parasite, suggests that Cry5B may have effects on other tissues in addition to the intestine.

### Ca^2+^ signaling and effects of Cry5B on the intestine

With message indicating the presence of glycosphingolipids receptors produced by BRE-5 activity and cadherin CDH-8 receptors, we sought to determine the time course and nature of the effects of Cry5B exposure on the *A*. *suum* intestine. We hypothesized that Cry5B would increase Ca^2+^ entry into the enterocytes, a feature seen with other pore forming toxins including phobalysin from *Photobacterium dameselae* [31]. Although, the effects of Cry5B have been studied on whole animals, the timing of direct effects on the enterocytes of the parasites were not known.

**Fig 3:**
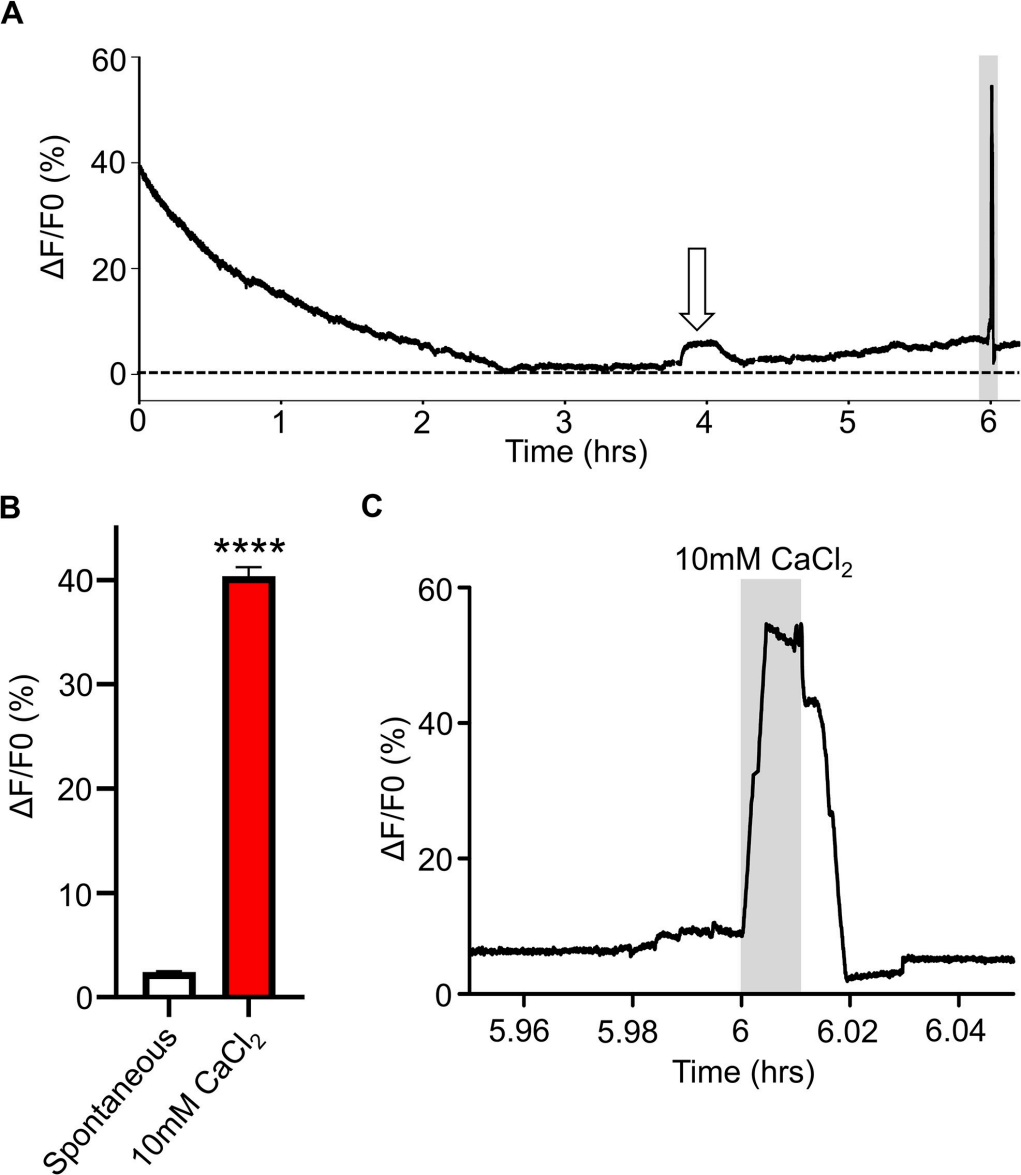
Long-term Ca^2+^ imaging. A) Representative trace of a 6-hour Ca^2+^ imaging recording on *A*. *suum* intestines in the presence of APF. White arrow highlights a spontaneous Ca^2+^ signal. Grey box indicates application of 10 mM CaCl_2_ a positive control. B) Amplitudes of spontaneous Ca^2+^ signals (white bar) and control 10 mM CaCl_2_ amplitudes (red bar). **** Significantly different to spontaneous. Spontaneous vs 10 mM CaCl_2_ *P* <0.0001 *t* = 41.76, *df* = 249, paired *t*-test, 5 individual female intestines. C) Close up of 10 mM CaCl_2_ signal from the end of the trace in A). Grey box highlights 10 mM CaCl_2_ application. *n* = 5 female intestines with a total of 250 of 250 regions providing viable responses which were used to generate the mean and SEM for B).

In control experiments, we found that we could maintain viable intestine tissues and perform Ca^2+^ imaging for at least 6 hours, Fig 3A. We observed a slow initial exponential decline in the relative Ca^2+^ fluorescence during the first two hours, a stable signal thereafter except for the occasional small spontaneous transient signals. We analyzed the largest transient signals during the 6-hour period and found the average amplitude of the spontaneous Ca^2+^ fluctuations were 2.4% ± 0.1%, Fig 3B, white bar. Although the spontaneous fluctuations indicate a healthy and viable tissue, as a positive control, we transiently increased the Ca^2+^ concentration by adding 10 mM CaCl_2_ to the bath after the 6-hour recording. As seen in Fig 3A and 3C, the pulse of 10 mM CaCl_2_ produced in a large increase in relative fluorescence that had a mean of 40.36% ± 0.88%, Fig 3B, red bar. When the Ca^2+^ was returned to 1 mM, the signal returned to the baseline, indicating that the tissue was not compromised. In damaged or deteriorating intestine preparations, the 10 mM CaCl_2_ test did not produce a Ca^2+^ signal increase nor a decline on washing with 1mM CaCl_2_ in APF.

**Fig 4:**
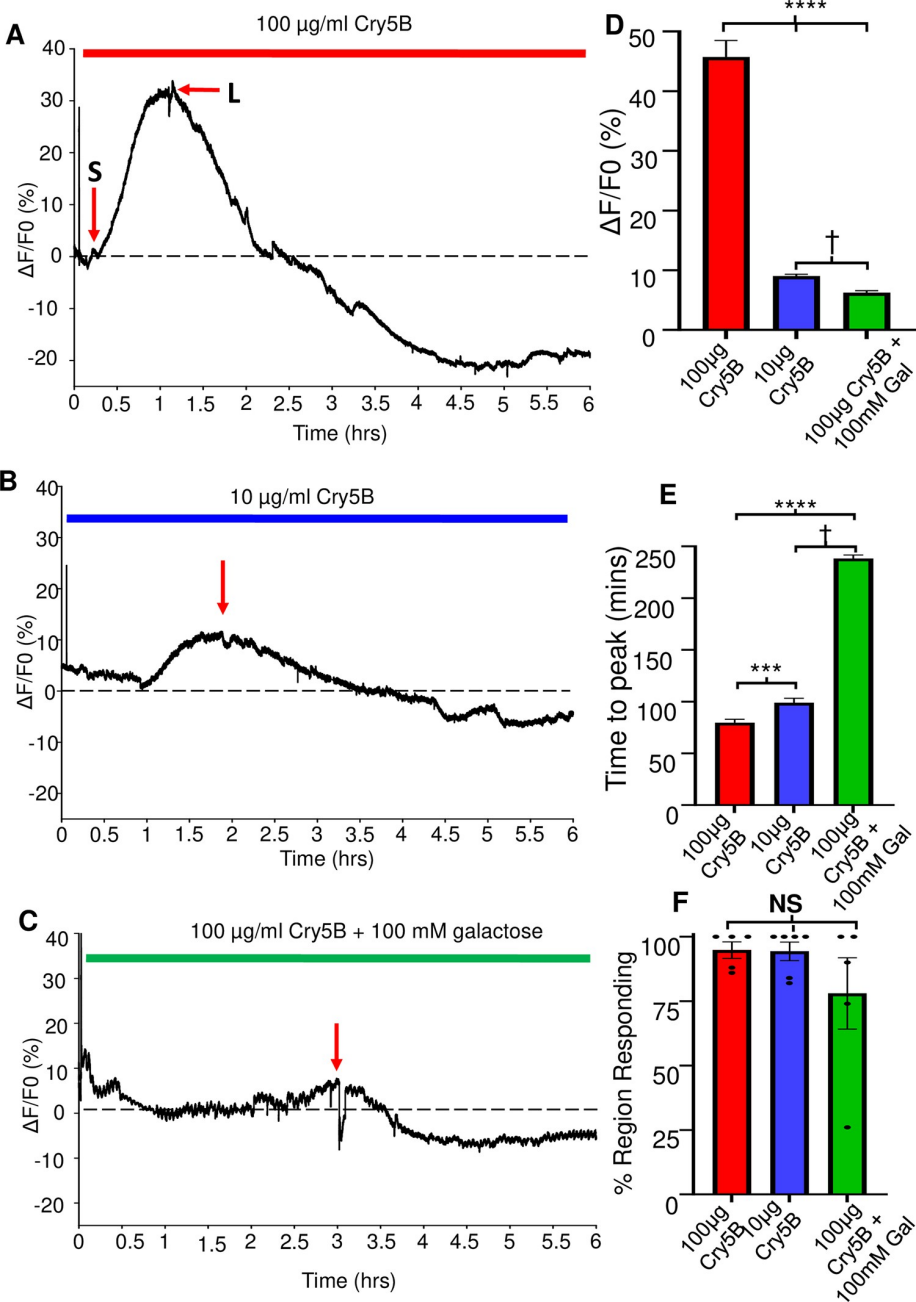
Cry5B stimulates concentration dependent Ca^2+^ peak and time-to-peak responses on *A*. *suum* intestines that are inhibited by galactose. A) Representative trace of a 6-hour Ca^2+^ recording to 100 μg/ml Cry5B. Dotted line highlights 0% F/F_0_. **S** indicates initial, small increase in Ca^2+^ and **L** represents the secondary larger response. B) Representative trace of a 6-hour Ca^2+^ recording to 10 μg/ml Cry5B. Dotted line highlights 0% F/F_0_. The red arrow indicates the peak of the Ca^2+^ signal. C) Representative trace of a 6-hour Ca^2+^ recording to 100 μg/ml Cry5B + 100 mM galactose. Dotted line highlights 0% F/F_0_. The red arrow indicates the peak of the Ca^2+^ signal. D) Average maximal amplitudes of Ca^2+^ fluorescence over 6 hours in response to 100 μg/ml Cry5B treated (red bar), 10 μg/ml Cry5B treated (blue bar) and 100 μg/ml Cry5B + 100 mM galactose treated (green bar). **** Significantly different to 100 μg/ml Cry5B. (100 μg/ml Cry5B vs 10 μg/ml Cry5B, *P* = <0.0001 *t* = 14.39, *df* = 518, unpaired *t*-test, 11 female intestines; 100 μg/ml Cry5B vs 100 μg/ml Cry5B + 100 mM galactose *P* = <0.0001 *t* = 14.55, *df* = 485, unpaired *t*-test; 10 female intestines). † Significantly different to 10 μg/ml Cry5B (10 μg/ml Cry5B vs 100 μg/ml Cry5B + 100 mM galactose *P* = <0.0001 *t* = 6.437, *df* = 531, unpaired *t*-test, 11 female intestines). E) Average time to maximal peak amplitude over 6 hours in response to 100 μg/ml Cry5B treated (red bar), 10 μg/ml Cry5B treated (blue bar) and 100 μg/ml Cry5B + 100 mM galactose treated (green bar). *** Significantly different to 100 μg/ml Cry5B. (100 μg/ml Cry5B vs 10 μg/ml Cry5B, *P* = <0.0007 *t* = 3.412, *df* = 518, unpaired *t*-test, 11 female intestines. **** Significantly different to 100 μg/ml Cry5B. 100 μg/ml Cry5B vs 100 μg/ml Cry5B + 100 mM galactose *P* = <0.0001 *t* = 14.55, *df* = 485, unpaired *t*-test, 10 female intestines). † Significantly different to 10 μg/ml Cry5B (10 μg/ml Cry5B vs 100 μg/ml Cry5B + 100 mM galactose *P* = <0.0001 *t* = 6.437, *df* = 531, unpaired *t*-test, 11 female intestines). F) % Region Responses: Mean % of regions showing Ca^2+^ responses bigger than the average spontaneous amplitude in untreated intestines in: red bar, 100 μg/ml Cry5B treated; blue bar, 10 μg/ml Cry5B treated; and green bar, 100 μg/ml Cry5B + 100 mM galactose treated. N.S. not significant compared to 100 μg/ml Cry5B (100 μg/ml Cry5B vs 10 μg/ml Cry5B, *P* = <0.9264 *t* = 0.095, *df* = 9, unpaired *t*-test, 11 female intestines; 100 μg/ml Cry5B vs 100 μg/ml Cry5B + 100 mM galactose *P* = <0.271 *t* = 1.183, *df* = 8, unpaired *t*-test, 10 female intestines). 100 μg/ml Cry5B *n* = 5 female intestines with 237 of 250 regions providing viable responses which were used to generate the mean and SEM for D & E; 13 of the 250 regions showed no response. 10 μg/ml Cry5B *n* = 6 female intestines with 283 of 300 regions produced responses which were used to generate the mean and SEM for D & E; 17 of 300 regions showed no response. 100 μg/ml Cry5B + 100 mM galactose *n* = 5 female intestines with 195 of 250 regions produced viable responses which were used to generate the mean and SEM for D & E, 55 of their 250 regions showed no response.

Initially, we tested a concentration of 100 μg/ml Cry5B on the intestine, based on whole animal *in vitro* studies on *A*. *suum* [16,32]. These studies reported that 100 μg/ml Cry5B was the *EC*_*50*_ concentration for *A*. *suum* being killed before 24 hours. Application of Cry5B to the intestines produced no immediate effects on the Ca^2+^ signal, Fig 4A. After one hour we observed small fluctuations in the signals which were followed by larger increases in fluorescence with an average maximal amplitude of 45.73% ± 2.8%, Fig 4D: red bar. The peak Ca^2+^ increases, which lasted around two hours, were seen at an average of 80 mins ± 3.3 mins, Fig 4E: red bar. After the peak, the Ca^2+^ signal declined slowly in a jerky manner below the zero level as the integrity of the enterocytes and intensity of the Fluo-3 fluorescence was lost which was observable down the microscope. The large Cry5B Ca^2+^ peak responses occurred in most of the 50 regions of the recorded intestines, 94.8% ± 3.2%, Fig 4F: red bar, and suggests that the distribution of the Cry5B receptors is widely and evenly distributed over the area of the intestine. Thus, we observed that the Ca^2+^ signals on the intestine of 100 μg/ml Cry5B are slow in onset (average peak 80 mins) but they are bigger than the levamisole and diethylcarbamazine anthelmintic compounds that we have tested previously [25,26].

### Histology

**Fig 5:**
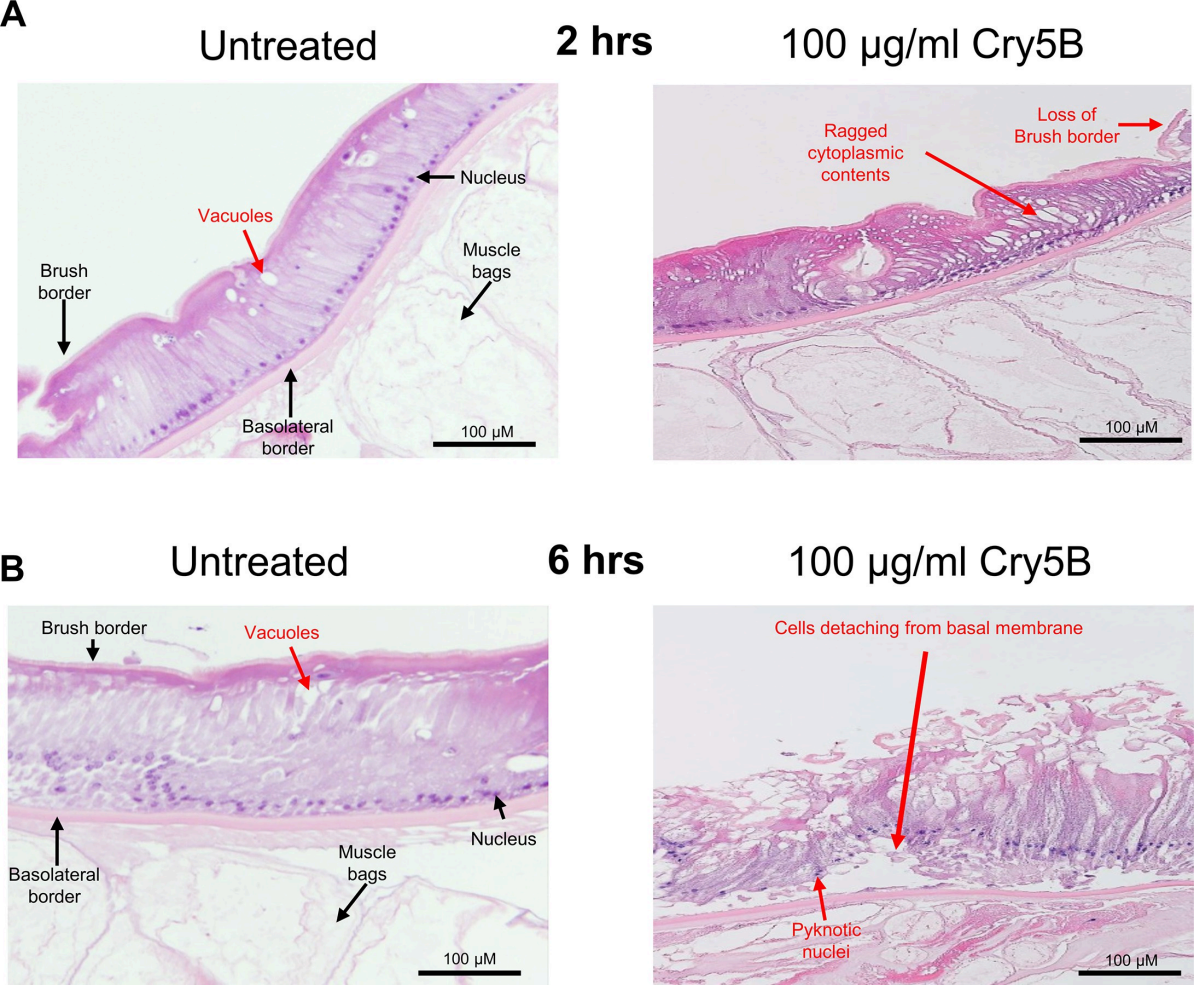
100 μg/ml Cry5B causes severe damage to the intestine of *A*. *suum* over 6 hours. Transverse H & E sections of the *Ascaris* intestine and underlying muscle bags in untreated (APF) *left column* and 100 μg/ml Cry5B treated *right column* at different time courses. Major structures and evidence of tissue damage are highlighted. A) 2hrs of treatment. B) 6hrs of treatment. After 6 hrs. there is marked degeneration of the epithelium with detachment of cells from the basal membrane.

With Cry5B producing large Ca^2+^ signals in the intestine, we evaluated how the Ca^2+^ signal correlated with histological effects on the intestine. We incubated body *A*. *suum* flap preparations that were cut along one lateral line through into the intestine in APF (untreated) or in APF containing 100 μg/ml Cry5B. We used the body flaps with the attached intestine to maintain the intestine position and to prevent folding. At the 2-hour time, where we observed the biggest Ca^2+^ signal amplitude in response to 100 μg/ml Cry5B, the consolidation of the cells was evident with coalescing vacuoles and loss of junctions in adjacent cells. Fig 5A: right panel. The untreated control sections at 2-hours, Fig 5A: left panel, did show some round vacuoles at the apical surface of the enterocytes, but the individual enterocytes did not show initial signs of separation between themselves and the vacuolation was more limited. The differences between the treated and untreated 2-hour sections were noticeable. After 6-hours, the fragmentation of the brush border was evident in 100 μg/ml Cry5B treated samples. The epithelial cells began to detach from the basolateral border and the nuclei appear to degenerate with pyknotic nuclei (Fig 5B: right panel). The untreated 6-hour section showed increased vacuolation, but the brush boarder was still intact, and enterocytes were not detached from the basolateral membrane, Fig 5B: left panel. Taken together, the histology demonstrates a direct effect of activated Cry5B on the intestine and the damage on this tissue that correlates with the Ca^2+^ signal. We have been able to establish a time of the 100 μg/ml Cry5B action that starts at around 1 hour and results in destruction of the integrity of the enterocytes in less than 6 hours. There is loss of the brush boarder, significant vacuolation and separation of adjacent enterocytes from the basolateral border, degeneration of the nuclei and cell death of the enterocytes that will damage the barrier properties of the intestine.

### Concentration-dependent effects of Cry5B

We have seen that 100 μg/ml Cry5B causes a large slow transient increase in the Ca^2+^ signal and histological damage to the intestine. Urban et al. 2013 [16], showed that low concentrations of Cry5B can still kill parasites but the incubation times are longer. We tested a concentration of 10 μg/ml Cry5B, Fig 4B and 4D: blue bar. The lower concentrations of Cry5B, still initiated Ca^2+^ signals, but they were significantly smaller than responses produced by 100 μg/ml Cry5B. The average amplitude of the 10 μg/ml Cry5B signals was 9.05% ± 0.3% Fig 4D: blue bar. The time-to-peak for 10 μg/ml Cry5B treated samples was 99 mins ± 4.4 mins, Fig 4E: blue bar, on average and was significantly slower than that of 100 μg/ml Cry5B. Again, after the peak the Ca^2+^ signal decreased below the zero level. Although the time-to-peak was on average smaller and slower to develop for 10 μg/ml Cry5B, we observed that the effects were still widespread and covered 94.3% ± 3.6% of the 50 observed regions in the different preparations during the recording, Fig 4F: blue bar.

**Fig 6:**
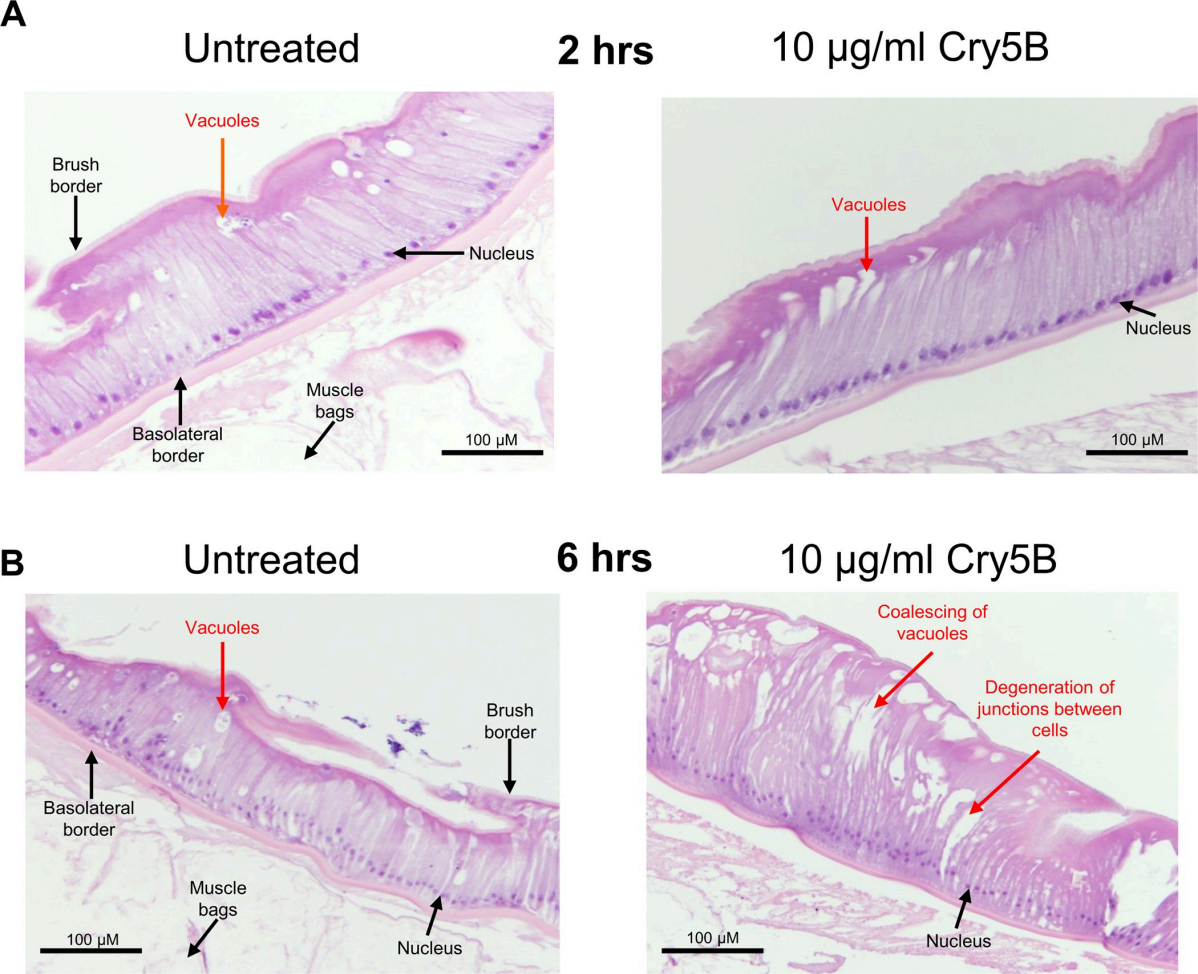
10 μg/ml Cry5B causes damage to the intestine of *A*. *suum* over 6 hours. Transverse H & E sections of the *A*. *suum* intestine and overlying muscle bags in untreated (APF) *left column* and 10 μg/ml Cry5B treated *right column*. Major structures and evidence of tissue damage are highlighted. Note the degeneration of junctions between cells and the coalescing vacuoles. A) After 2hrs of treatment. B) After 6hrs of treatment.

We tested the effects of 10 μg/ml Cry5B for 2-hours and for 6-hours on the histology sections of the intestines and observed changes that were less severe. At the 2-hour time point nearer the peak of the Ca^2+^ signal, we observed differences between the untreated and 10 μg/ml Cry5B treated preparations (Fig 6A). There were larger elongated vacuoles occurring in the Cry5B treated samples near the apical region of the enterocytes and some separation between adjacent enterocytes: these sections contrasted with those of the 2-hour untreated sections, (Fig 6A; left panel), which showed no separation between enterocytes and smaller round vacuoles. The 6-hour 10 μg/ml Cry5B sections had more coalescing of vacuoles which were larger in the treated sections compared to the untreated sections, increased degeneration of junctions between cells, and there was some erosion of parts of the brush border, Fig 6B. We concluded that the effects of Cry5B on Ca^2+^ signaling and histology on the intestine were concentration-dependent, with 10 μg/ml Cry5B producing smaller and slower Ca^2+^ peak amplitudes with damage occurring at a slower rate than with the 100 μg/ml. However, both 10 μg/ml and 100 μg/ml had effects on nearly all the 50 regions of the intestines; and the 6-hour time is sufficient for activated toxin to damage to the entire intestinal tissue.

### Galactose inhibits the action of Cry5B

The action of Cry5B on nematodes has included interaction with glycosphingolipid receptors produced by BRE-5 [12,18–20,30,33] that 100 mM galactose inhibits the action of Cry5B by competing for binding to the glycosphingolipid and promotes survivability long-term. We have identified the *A*. *suum* orthologue for *bre-5* is present in the intestine, Fig 2A, suggesting that the observed Cry5B stimulated Ca^2+^ signal and subsequent intestinal damage involves interaction with BRE-5 produced glycosphingolipids. We tested the effects of 100 mM galactose on the effects of 100 μg/ml Cry5B on the Ca^2+^ signal and histology of the intestine.

**Fig 7:**
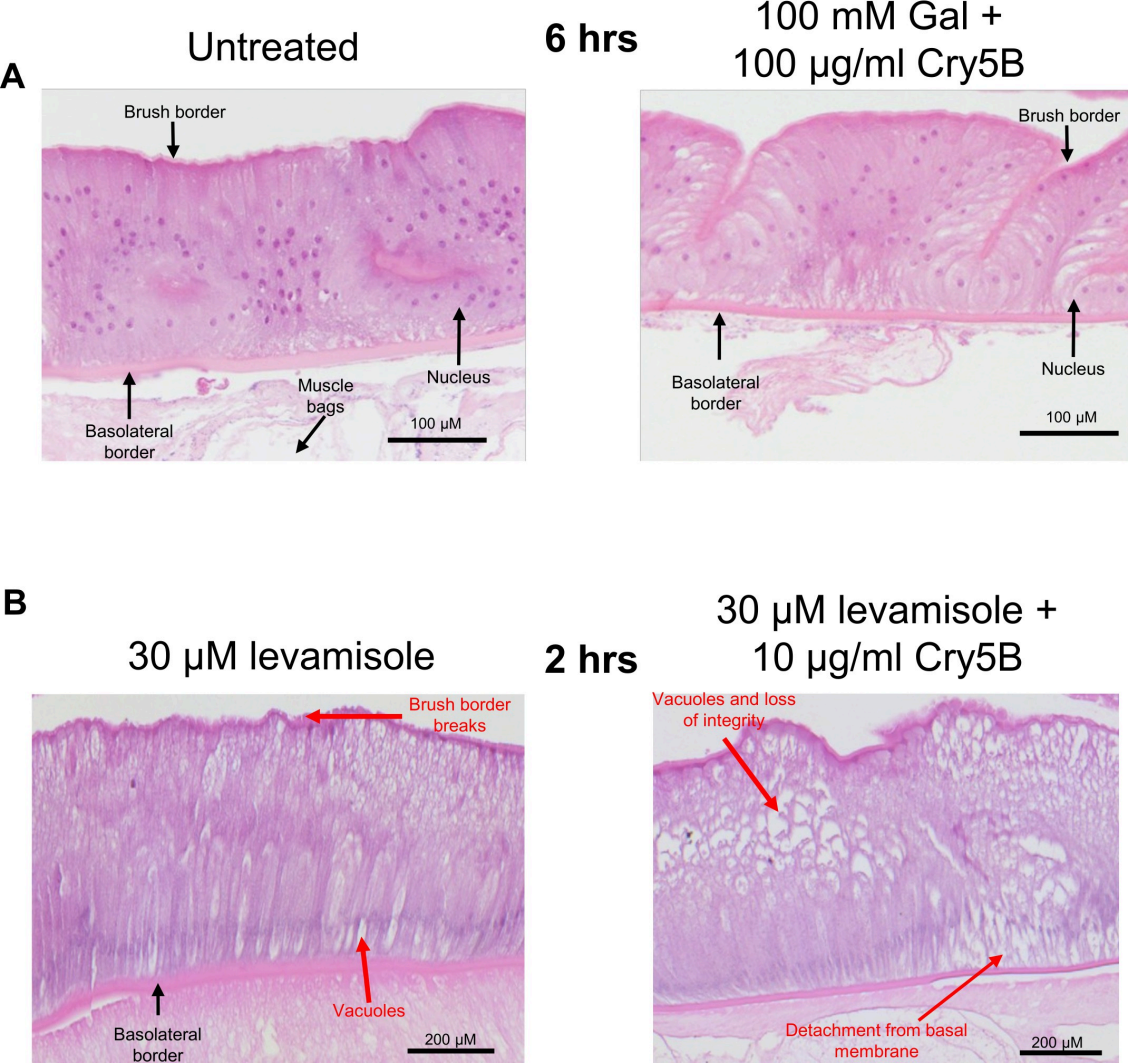
Histological effects of 100 μg/ml Cry5B + 100 mM galactose and 30 μM levamisole + 10 μg/ml Cry5B. A) 100 mM galactose prevents intestinal damage caused by 100 μg/ml Cry5B. Transverse H & E sections of the *A*. *suum* intestine and underlying muscle bags in untreated (APF) *left column* and 100 μg/ml Cry5B + 100 mM galactose treated *right column* after 6hrs. of treatment. Major structures are highlighted. Note the loss of attached muscle cell bags in 100 μg/ml Cry5B + 100 mM galactose treated example is due to mechanical damage during preparation. B) Transverse H & E sections of the *A*. *suum* intestine and underlying muscle bags after 2 hours in 30 μM levamisole (left panel) and 30 μM levamisole + 10 μg/ml Cry5B (right panel). Major structures and evidence of tissue damage are highlighted. Treatment with both levamisole alone and levamisole + Cry5B resulted in the loss of recognizable nuclei. Addition of 10 μg/ml Cry5B and 30 μM levamisole resulted in significant loss of cell integrity compared to 30 μM levamisole or 10 μg/ml Cry5B alone. While levamisole alone caused subtle changes to epithelial cells, levamisole + Cry5B treatment resulted in condensation of the cytoplasm and ragged cellular contents.

100 mM galactose strikingly inhibited in the Ca^2+^ signal produced by Cry5B, Fig 4A and 4C. The increase in the 100 μg/ml Cry5B Ca^2+^ signal in the presence of 100 mM galactose was significantly smaller than either the 100 μg/ml Cry5B or the 10 μg/ml Cry5B, Fig 4D: green bar. Additionally, the average time-to-peak was significantly longer than either the 100 μg/ml Cry5B and 10 μg/ml Cry5B, being 238 mins ± 3.4 mins, Fig 4E: green bar. Although the average percentage of the 50 regions in the different preparations showing a Ca^2+^ response larger than the spontaneous signals of the untreated intestines was reduced to 78% ± 13.84, Fig 4F: green bar, it did not reach statistical significance. Histology showed that in the presence of 100 mM galactose the Cry5B mediated damage to the intestines was significantly inhibited, even at 6-hours, Fig 7A. These observations suggest that the mode of action of Cry5B is conserved in the *A*. *suum* intestine and that Cry5B initiates intestinal damage that starts from the interaction with glycosphingolipid receptors. The competitive binding by galactose limits intestinal damage.

### Levamisole potentiates Cry5B Ca^2+^ signals

The cholinergic anthelmintic levamisole is used to treat nematode parasite infections. We have previously demonstrated that the subunits for the levamisole sensitive nicotinic acetylcholine receptor (nAChR) are expressed in the intestine of *A*. *suum*, and that application of levamisole generates a distinctive Ca^2+^ signal that is inhibited using mecamylamine [25]. Hu et al. 2010b [23] have observed that combination of levamisole and Cry5B has a synergistic killing effect on whole *C*. *elegans*.

**Fig 8:**
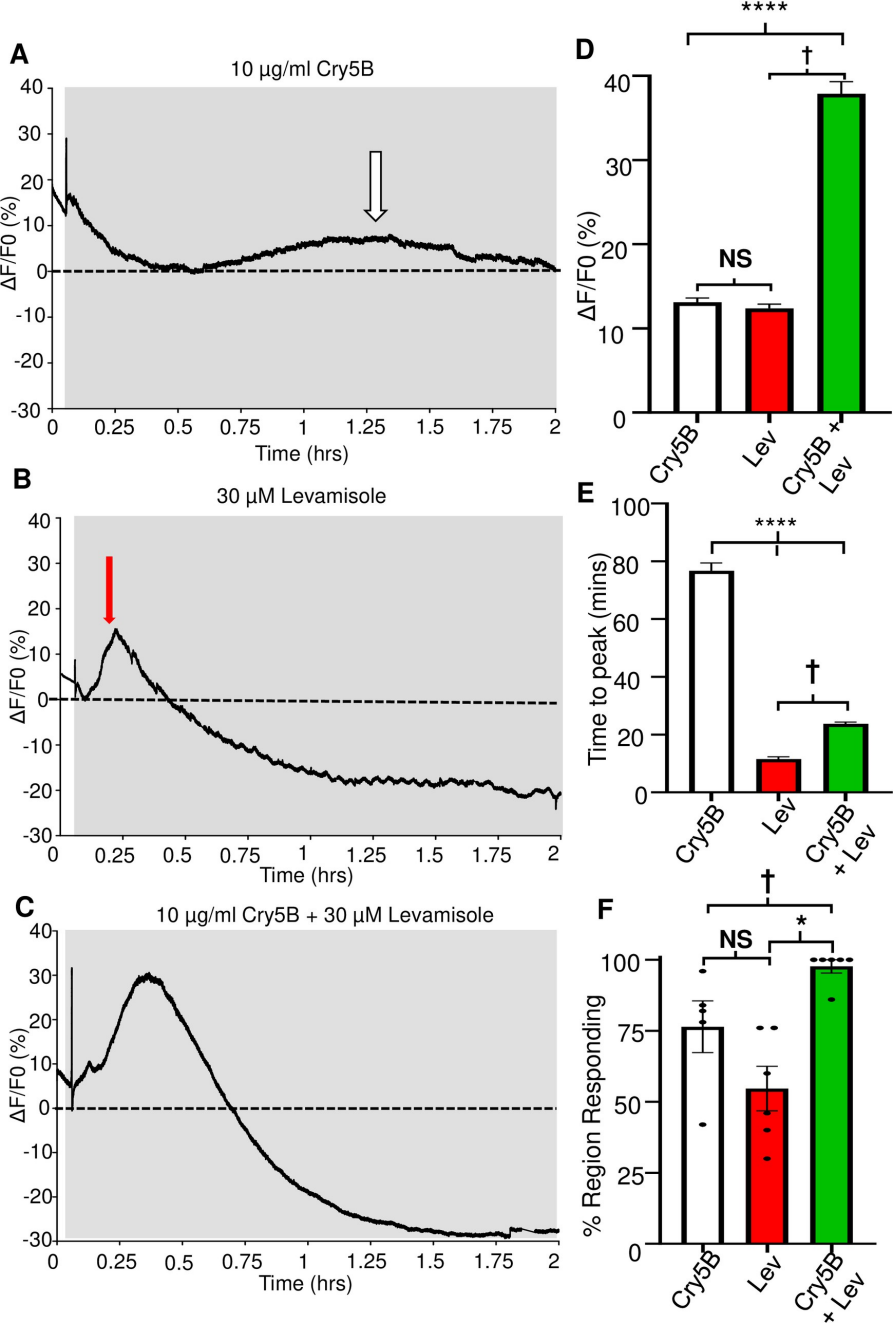
Levamisole potentiates Cry5B signal in *A*. *suum* intestines. A) Representative trace highlighting a 2-hour recording in response to 10 μg/ml Cry5B. Grey box indicates Cry5B application and dotted line represents 0% F/F_0_. White arrow indicates peak of the Ca^2+^ signal. B) Representative trace highlighting a 2-hour recording in response to 30 μM levamisole. Grey box indicates levamisole application and dotted line represents 0% F/F_0_. The red arrow indicates the peak of the Ca^2+^ signal. C) Representative trace highlighting a 2-hour recording in response to the combination of 10 μg/ml Cry5B and 30 μM levamisole. The grey box indicates Cry5B, and levamisole application and dotted line represents 0% F/F_0_. The red arrow highlights the initial levamisole response and green arrow indicates the larger secondary response. D) The white bar shows the mean maximal amplitudes of Ca^2+^ fluorescence over 2 hours in 10 μg/ml Cry5B treated. The red bar shows the 30 μM levamisole responses and the green bar shows the 10 μg/ml Cry5B + 30 μM levamisole responses. N.S. not significantly different to 10 μg/ml Cry5B (10 μg/ml Cry5B vs 30 μM levamisole, *P* = < 0.3117 *t* = 1.013, *df* = 393, unpaired *t*-test, 11 female intestines). **** Significantly different to 10 μg/ml Cry5B (10 μg/ml Cry5B vs 10 μg/ml Cry5B + 30 μM levamisole, *P* = < 0.0001 *t* = 13.58, *df* = 482, unpaired *t*-test, 12 female intestines). † Significantly different to 30 μM levamisole (30 μM levamisole vs 10 μg/ml Cry5B + 30 μM levamisole *P* = < 0.0001 *t* = 13.41, *df* = 465, unpaired *t*-test, 12 female intestines). E) Mean times-to-peak amplitude over 2 hours in 10 μg/ml Cry5B treated (white bar), 30 μM levamisole (red bar) and 10 μg/ml Cry5B + 30 μM levamisole (green bar). **** Significantly different to 10 μg/ml Cry5B (10 μg/ml Cry5B vs 30 μM levamisole, *P* = < 0.0001 *t* = 22.11, *df* = 363, unpaired *t*-test 11 female intestines; 10 μg/ml Cry5B vs 10 μg/ml Cry5B + 30 μM levamisole, *P* = < 0.0001 *t* = 23, *df* = 482 unpaired *t*-test, 12 female worms). † Significantly different to 30 μM levamisole (30 μM levamisole vs 10 μg/ml Cry5B + 30 μM levamisole *P* = < 0.0001 *t* = 12.37, *df* = 465, unpaired *t*-test, 12 female intestines). F) Mean percent of regions showing a Ca^2+^ response larger than the average spontaneous Ca^2+^ amplitude in untreated intestines in 10 μg/ml Cry5B treated (white bar), 30 μM levamisole (red bar) and 10 μg/ml Cry5B + 30 μM levamisole (green bar). N.S. not significant compared to 10 μg/ml Cry5B (10 μg/ml Cry5B vs 30 μM levamisole, *P* = < 0.102 *t* = 1.821, *df* = 9, unpaired *t*-test, 11 female intestines). * Significantly different to 10 μg/ml Cry5B (10 μg/ml Cry5B vs 10 μg/ml Cry5B + 30 μM levamisole *P* = < 0.0357 *t* = 2.468, *df* = 9, unpaired *t*-test, 11 female intestines). † Significantly different to 30 μM levamisole (30 μM levamisole vs 10 μg/ml Cry5B + 30 μM levamisole, *P* = < 0.0004 *t* = 5.266, *df* = 10, unpaired *t*-test, 12 female intestines). 10 μg/ml Cry5B was tested on *n* = 5 female intestines showed 191 out of 250 regions that produced viable responses that were used to generate the means and SEM for D & E; 59 of 250 regions showed no response. 30 μM levamisole was tested on *n* = 6 individual female intestines and 174 of 300 regions produced viable responses which were used to generate the mean and SEM for D & E, 126 of 300 regions showed no response. 10 μg/ml Cry5B + 30 μM levamisole was tested on *n* = 6 individual female worms that had 293 of 300 regions showing a viable response that were used to generate the mean and SEM for D & E, 7 of the 300 regions did not respond.

We sought to determine if this effect could be detected as an interaction between Cry5B and levamisole on the *A*. *suum* intestine using the approach that we have described above. 10 μg/ml Cry5B applied over 2-hours produced peak Ca^2+^ responses with amplitudes of 13.11% ± 0.51% like the 6-hour experiments with at an average time of 76.6 mins ± 2.7 mins, Fig 8A, 8D and 8E; white bars. We did, however, observe a reduction in the average % of the regions of the intestines responding to Cry5B for the 2 hours incubations compared to 6 hours, which went down to 76.4% ± 9.1%, Fig 8F: white bar, suggesting that shorter term incubations result in fewer regions of the intestine responding.

Application of 30 μM levamisole to the *A*. *suum* intestine produces an early, ~10 min sharp peak, with an average amplitude around 10% [25]. We incubated intestinal tissues over 2 hours and recorded the levamisole signal to look for any effects of 30 μM levamisole exposure that we had not seen with our previous shorter incubations [25]. The 2-hour levamisole Ca^2+^ responses were like those we had reported previously. The peak Ca^2+^ response had an average amplitude of 12.4% ± 0.5% and time-to-peak of 11.5 mins ± 0.8 mins, Fig 8B and 8D: red bar and 8E: red bar. After the initial peak, we saw no further major peaks other than small oscillations in the signal for the rest of the recording, Fig 8B. These observations show that levamisole causes a more rapid influx of Ca^2+^ into the intestinal enterocytes than Cry5B. The levamisole mediated Ca^2+^ signal distribution was limited to 58% ± 7% of the intestine, Fig 8F: red bar, suggesting that the levamisole receptors are not evenly distributed over the surface of the intestine.

With both 10 μg/ml Cry5B and 30 μM levamisole producing similar peak amplitudes, ~12%, but having different times-to-peak (11 mins vs 77 mins) we sought to determine if co-application of the two compounds altered the Ca^2+^ signal and/or affected the number of regions responding. Significantly, the combination of the two compounds resulted in a larger Ca^2+^ signal that had an average peak amplitude of 37.9% ± 1.4%, Fig 8C and 8D: green bar, which took a significantly shorter time-to-peak, 23.8 mins ± 0.6 mins, Fig 8E: green bar compared to Cry5B alone. Although slower than the levamisole alone, this larger signal reveals the effect of their combination. Additionally, the combination of both 10 μg/ml Cry5B and levamisole resulted in 97.7% ± 2.3% of the regions tested showing a response, Fig 8F: green bar, showing that the combination affects the intestine across more of the areas than either of the compounds used alone.

### Mecamylamine inhibits the synergistic effect of levamisole on the Cry5B response

**Fig 9:**
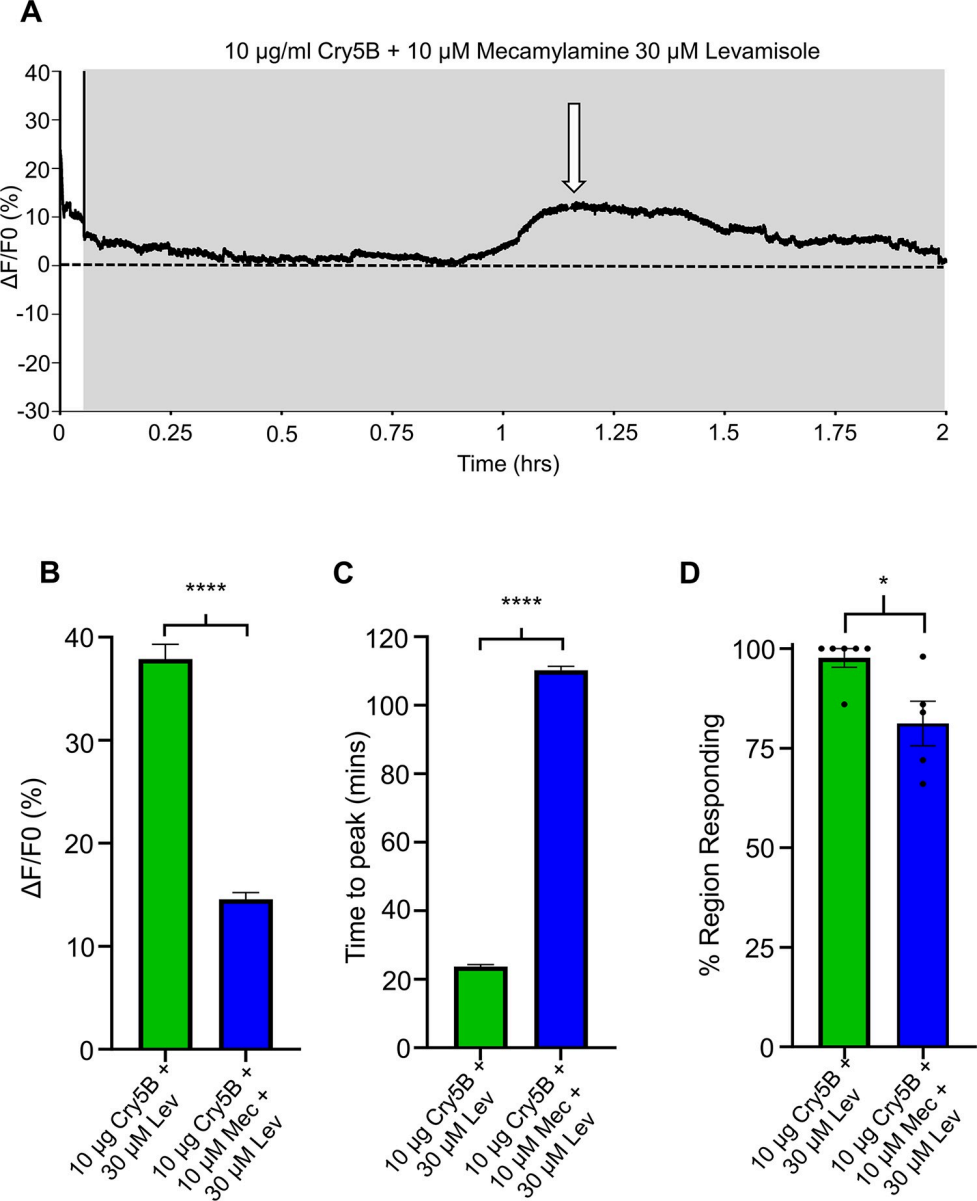
Mecamylamine inhibits levamisole mediated potentiation Cry5B. A) Representative trace highlighting a 2-hour recording in response to 10 μM mecamylamine, 30 μM levamisole and 10 μg/ml Cry5B. The grey box shows the co-application of mecamylamine, levamisole and Cry5B. The dotted line represents 0% F/F_0_. White arrow indicates the peak of the Ca^2+^ signal. B) Average maximal amplitudes of Ca^2+^ fluorescence over 2 hours in 30 μM levamisole + 10 μg/ml Cry5B treated (green bar) and 10 μM mecamylamine + 30 μM levamisole + 10 μg/ml Cry5B (blue bar) treated intestines. **** Significantly different: 30 μM levamisole+10 μg/ml Cry5B *vs* 10 μM mecamylamine+30 μM levamisole+10 μg/ml Cry5B (*P* = < 0.0001 *t* = 12.92, *df* = 494, unpaired *t*-test, 11 female intestines). C) Average time to maximal peak amplitude over 2 hours in 30 μM levamisole+10 μg/ml Cry5B treated (green bar) and 10 μM mecamylamine+30 μM levamisole+10 μg/ml Cry5B (blue bar) treated intestines. **** Significantly different: 30 μM levamisole+10 μg/ml Cry5B vs 10 μM mecamylamine+30 μM levamisole+10 μg/ml Cry5B (*P* = < 0.0001 *t* = 72.6, *df* = 494, unpaired *t*-test, 11 female intestines). D) Average percent of regions showing a Ca^2+^ response larger than the average spontaneous Ca^2+^ amplitude in untreated intestines. 30 μM levamisole+10 μg/ml Cry5B treated (green bar) and 10 μM mecamylamine+30 μM levamisole+10 μg/ml Cry5B (blue bar). * Significantly different: 30 μM levamisole+10 μg/ml Cry5B vs 10 μM mecamylamine+30 μM levamisole+10 μg/ml Cry5B (*P* = < 0.0176, *t* = 2.899, *df* = 9, unpaired *t*-test, 11 female intestines). 10 μg/ml Cry5B+30 μM levamisole *n* = 6 individual female worms with a total of 293 of 300 regions providing a viable response that were used to generate the mean and SEM for B & C. Note: 7 of 300 regions showed no response. 10 μM mecamylamine+30 μM levamisole+10 μg/ml Cry5B *n =* 5 individual female worms with a total of 203 of 250 providing a viable response to generate the mean and SEM for B & C. Note that 47 of 250 showed no response.

To determine if the stronger and faster signals observed for the levamisole and Cry5B combination was caused by levamisole interaction on the L-type nAChR, we used mecamylamine, which we have previously demonstrated is able to inhibit the levamisole mediated Ca^2+^ signals [25]. We pre-treated intestinal preparations with 10 μM mecamylamine before adding a triple combination of 10 μM mecamylamine, 30 μM levamisole and 10 μg/ml Cry5B. We observed that the combination effect was inhibited in the presence of mecamylamine, Fig 9A. The average Ca^2+^ amplitude was reduced to 14.56% ± 0.64%, which is significantly smaller than the combination of levamisole + Cry5B, Fig 9B: blue bar. The time-to-peak of 100.2 mins ± 1.16mins was also significantly longer, Fig 9C: blue bar. We observed the number of regions providing a positive response was also significantly reduced in the presence of mecamylamine, with an average of 81.2% ± 5.6%, Fig 9D: blue bar. Our results highlight that the combination of both Cry5B, and levamisole causes faster and bigger Ca^2+^ responses over larger areas of the intestine compared to use of the either compound alone; and that this synergism is mediated by levamisole interacting with L-type nAChRs that are inhibited by the selective nicotinic cholinergic antagonist, mecamylamine.

### Histological effects of the levamisole and Cry5B combination

As before, we treated intestines attached to our *A*. *suum* body flaps over 2 hours in either 30 μM levamisole or 30 μM levamisole + 10 μg/ml Cry5B, and prepared histological slides to observe structural effects. The 2-hour 30 μM levamisole treated sample did show a level of change in the enterocytes. Fig 7B; left panel, shows that treatment with levamisole caused vacuolation and some disruption throughout the intestine, but the vacuoles were more distributed and more apparent at the apical region of the cells. There was separation between some of the neighboring cells at the basolateral region of the preparation. We also observed evidence of damage to the brush border of the intestine, with small breaks appearing along the apical region of the enterocytes. These observations suggest that levamisole, on its own, can disrupt the integrity of the intestine with some unique phenotypes, illustrating a mode of action for this compound other than on the neuromuscular system of the nematode parasites.

Although levamisole and Cry5B alone cause some degree of intestinal damage, the combination of the two compounds resulted in more severe destruction. As shown in Fig 7B; right panel, the enterocytes of intestines that were treated with both compounds showed more severe damage, with more vacuoles, more tearing and loss of integrity throughout the intestine, detachment from the basolateral membrane and increased evidence of necrotic cells. The increased histological damage is correlated with the bigger and faster Ca^2+^ signal suggesting that the enterocytes are showing necrotic cell-death at a faster rate than when either compound is used alone. The combination of Cry5B and the cholinergic anthelmintic levamisole leads to faster destruction of the *A*. *suum* intestine that will contribute to a faster killing of the nematode parasites [16].

## Discussion

### Cry5B, Ca^2+^ and necrosis

The arthroseries glycolipids and CDH-8 have been described as Cry5B receptors in *C*. *elegans* [18–21]. We observed that message for *bre-5* [18–20] required for the synthesis of arthroseries glycolipids, and *cdh-8* message required for the synthesis of cadherin [21], were present in the *A*. *suum* intestine. Once Cry5B has bound to its receptors, it oligomerizes and forms small 1–2 nm pores in the plasma membrane, which based on analogy to Cry1Aa and Cry1Ac pores, are permeable to Ca^2+^ [34]. We found that Cry5B produced a slow, but large, dose-dependent increase in the Ca^2+^ signal that peaked at an average of 80 minutes across 95% of the regions of intestine. The effects of Cry5B were inhibited by 100mM galactose which inhibits Cry5B binding to the glycolipids [18,19] suggesting the arthroseries glycolipids, rather than the cadherins are the major receptor targets in *A*. *suum* intestines.

Histology showed significant damage produced by Cry5B at 2 hours and severe necrosis by 6 hours. The damage included vacuolation in the enterocytes, damage to the enterocyte brush borders, separations between adjacent enterocytes and pyknotic nuclei. These effects may be explained by a Ca^2+^ mediated necrosis cell-death pathway, Fig 9, that follows Ca^2+^ overload due to Ca^2+^ entry through Cry5B toxin pores formed in the plasma membranes of the enterocytes [21,35].

### Necrosis Cell-Death Pathway

In the *A*. *suum* intestines we have seen that there is necrosis and death of the enterocytes associated with the maintained rise in cytosolic Ca^2+^. In *C*. *elegans*, an excessive and uncontrolled increase in cytosolic Ca^2+^ in enterocytes has been described [35] that leads to the necrosis cell-death pathway. The rise in Ca^2+^ produces rupture of the lysosomes, release of cathepsins and calpains and then the necrosis cascade and cell-death pathway [36–38]. This pathway that is driven by increased cytosolic Ca^2+^ will be affected by: 1) uptake by plasma and sarcoplasmic reticulum ATPases and the Ca^2+^ exchangers; 2) release from ryanodine receptors, release from IP3 receptors, and release from Ca^2+^ binding proteins; and 3) entry through the plasma membrane by routes that may include diethylcarbamazine-sensitive TRP channels [26], levamisole activated L-AChR channels [25] and bacterial pores like Cry5B. Once the cytosolic Ca^2+^ exceeds a sustained critical level the necrosis cell-death pathway becomes irreversible and leads to destruction of the intestine and then the nematode.

### Defense pathways against Cry5B

If the Ca^2+^ does not exceed the sustained critical level, the enterocytes can respond against this attack by activating defense pathways. Low concentrations of Cry5B, although they open Ca^2+^ permeable cation pores in cell plasma membranes, they may not kill *C*. *elegans* or nematode parasites. Nematodes can resist the effects of Cry5B by activating intrinsic cellular defense (INCED) pathways [39–43].

### Levamisole

**Fig 10:**
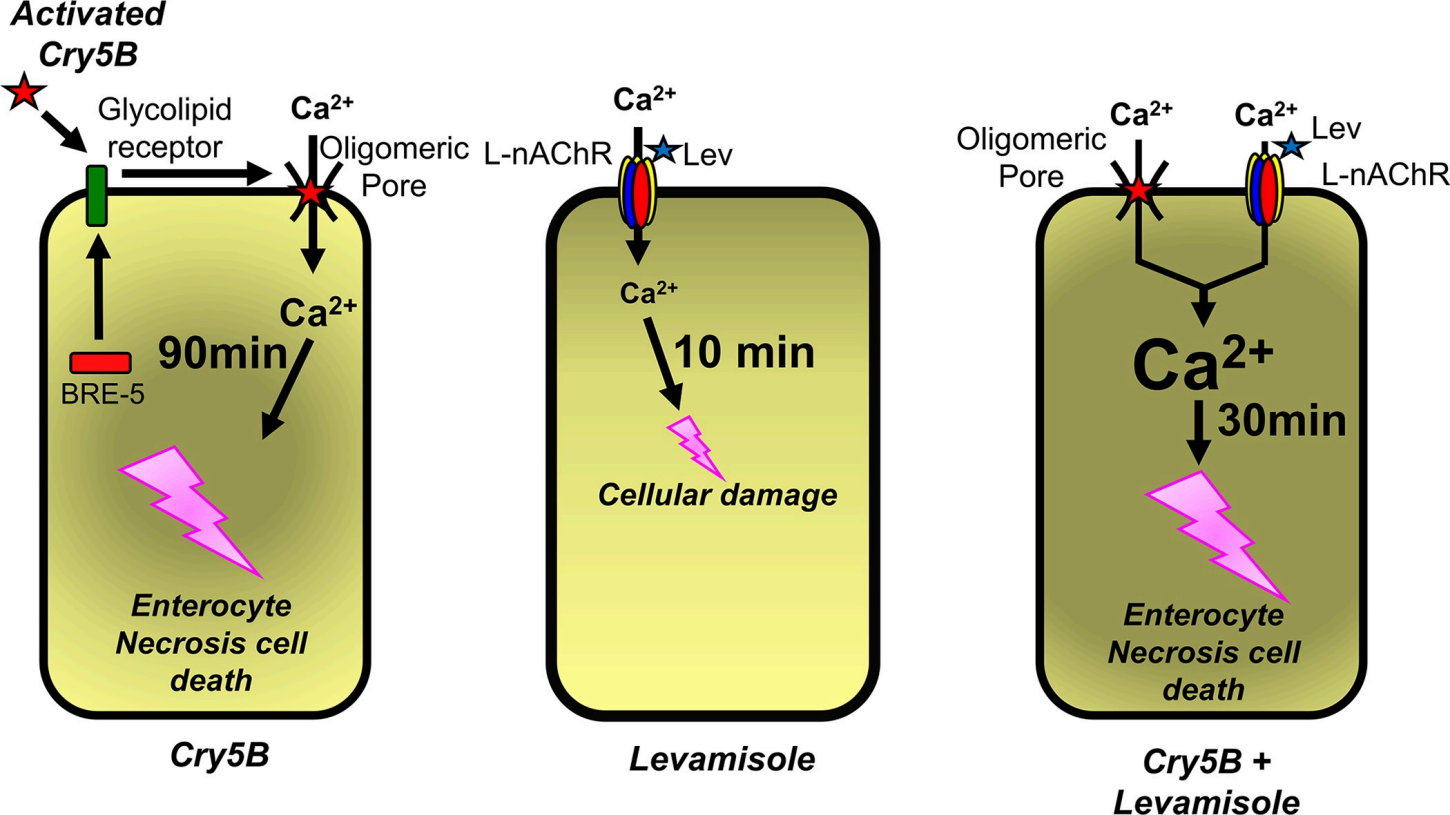
Summary diagram of proposed mode of action of Cry5B and levamisole action on the intestine of *Ascaris suum* precipitating necrotic cell-death. BRE-5 produced glycosphingolipids are receptors for activated Cry5B. Following binding to the glycolipid receptors, Cry5B forms oligomeric pores in the plasma membrane. These activated pores are permeable to Ca^2+^ that gives rise to an increase in cytosolic calcium over a period of 90 minutes. This rise in cytosolic Ca^2+^ exceeds the sustained critical level, so that the necrosis cell-death pathway becomes irreversible and leads to histological destruction of the intestine and then the nematode. Levamisole activates L-subtype nAChR channels that are Ca^2+^ permeable and if sustained, will lead to histological damage of the intestine too. The combination of levamisole and Cry5B, both of which produce a rise in cytosolic Ca^2+^ in the enterocytes produces a faster and bigger rise in cytosolic Ca^2+^ and a more rapid onset of the necrosis cell-death pathway.

Levamisole is an agonist of nematode Ca^2+^ permeable L-AChR ion-channels [44] which are expressed in the *A*. *suum* intestines [25] as well as muscle [45]. Levamisole produces a distinctive sharp Ca^2+^ peak in the enterocytes, at an average of 12 minutes after application to the intestine [25] but which is limited to 58% of the area of the intestine. This peak at 12 minutes is much slower than that which follows levamisole application to body muscle: it may therefore be due to a secondary Ca^2+^ release that follows opening of enterocyte L-type AChRs [25]. Nonetheless, the application of 10 μg/mg Cry5B and levamisole together shortened the onset time to the Cry5B peak and resulted in a larger Ca^2+^ peak signal that was seen at an average of 24 minutes across nearly all the areas of the intestines. The Cry5B peak was slower than the levamisole peak but was much bigger than the 10 μg/ml Cry5B alone. The potentiating effects of levamisole on the effects of Cry5B are underpinned by these two agents acting on two separate Ca^2+^ permeable channels in the plasma membranes, both of which increase the calcium concentration in the enterocytes to produce cytotoxicity. Fig 10.

### Defense pathways against levamisole

Little is known about the effects of maintained concentrations of levamisole on nematode intestines but studies on the effects of maintained concentrations of levamisole on nematode muscle have been observed in *Brugia malayi* [45] and *C*. *elegans* [46]. The effects of maintained concentrations of levamisole following spastic paralysis leads to flaccid paralysis and then a recovery of motility through processes that allows for homeostatic plasticity (accommodation and adjustment to maintained stimuli). In *B*. *malayi* there is a loss of functional L-AChR channels an increase in levamisole resistant AChRs, and a decrease in *nra-2* (a nicalin homologue that encodes an ER retention protein) that allows faster loss of sensitivity to levamisole and recovery. The regulators of desensitization and therefore defense and recovery of AChRs that are present in *C*. *elegans* muscle and *Brugia* include: TAX-6, a calcineurin A subunit, that affect desensitization of AChRs in rat chromaffin cells; SOC-1 (a multi-subunit adaptor protein); and PLK-2 (a serine/threonine kinase) [47–49].

### How do the levamisole and Cry5B interact?

In *C*. *elegans* it is not known if the enterocytes possess levamisole sensitive nAChRs, but it is known that there is a synergistic interaction between levamisole and Cry5B that displays hyper-susceptibility on the whole worm [22]. In *A*. *suum* there are levamisole-sensitive L-subtype nAChRs on the enterocytes [25] and evidence of Cry5 glycolipid receptors: activation of theses nAChRs receptors leads to increased cytoplasmic Ca^2+^. If either Cry5B or levamisole induces a cytoplasmic Ca^2+^ near a critical level, then the addition of the other agent will trigger the necrosis cell-death pathway, Fig 10. If the cytoplasmic Ca^2+^ levels are not sufficient to trigger the necrosis cell-death pathway, the defense pathways will permit the enterocytes to recover and overcome the effects of the Cry5B and levamisole. Based on our observations there is a direct synergistic action of levamisole and Cry5B on the intestine. On the whole worm the destruction of the protective barrier provided by the intestine, levamisole could then have easier access to nAChRs on the underlying muscles. With the identification of Cry5B targets in the body wall, there is also the potential of additional synergistic actions on muscle and nerves in addition to that we have seen on the intestine.

## Conclusion

We have observed that levamisole and Cry5B produce an increase in Ca^2+^ in the enterocytes of the *A*. *suum* intestine and lead to necrosis and cell-death. The effects of the two compounds are synergistic, being much bigger when both are administered together. A combination of the two compounds is expected to be more effective than either of the two compounds alone and be more effective in the presence of resistance to one of these compounds.

## Supporting information

S1 Fig Localization of Cry5B targets in the intestine and muscle bag region. Original uncropped gel pictures from Fig 2 showing RT-PCR analysis of intestine (1i, 2i, 3i, 4i, 5i) and muscle bag (1b, 2b, 3b, 4b, 5b) of five separate female *A*. *suum* worms. Each lane represents the intestine or muscle bag of an individual worm. *Asu-gapdh* from the intestine (Ci) or muscle bag (Cb) was used as a positive control. N.C. = negative control, no cDNA template present. M = FastRuler Middle Range DNA Ladder (ThermoFisher Scientific). A) *Asu-bre-5*, B) *Asu-cdh-8* Images were taken under UV light with an exposure setting of 3 seconds per 1 frame.
(TIF)

S1 Table Primer sequences used for RT-PCR. Forward and reverse primer sequences for *bre-5*, *cdh-8* and the reference gene *gapdh* in *Ascaris suum*. (TIF)

S2 Table Primer sequences targeting middle of each gene for quantitative PCR. Forward and reverse primers used for qPCR experiments for *bre-5*, *cdh-8* and the reference gene *gapdh* in *Ascaris suum*. (TIF)

S3 Table Accession numbers for *asu-bre-5* and *Asu-cdh-8*. (TIF)
