# Supplementary figures and images for: The nematode (Ascaris suum) intestine is a location of synergistic anthelmintic effects of Cry5B and levamisole
Source: PLoS Pathog. 2024 May 17;20(5):e1011835. doi: 10.1371/journal.ppat.1011835 (PMC11139322; doi:10.1371/journal.ppat.1011835)

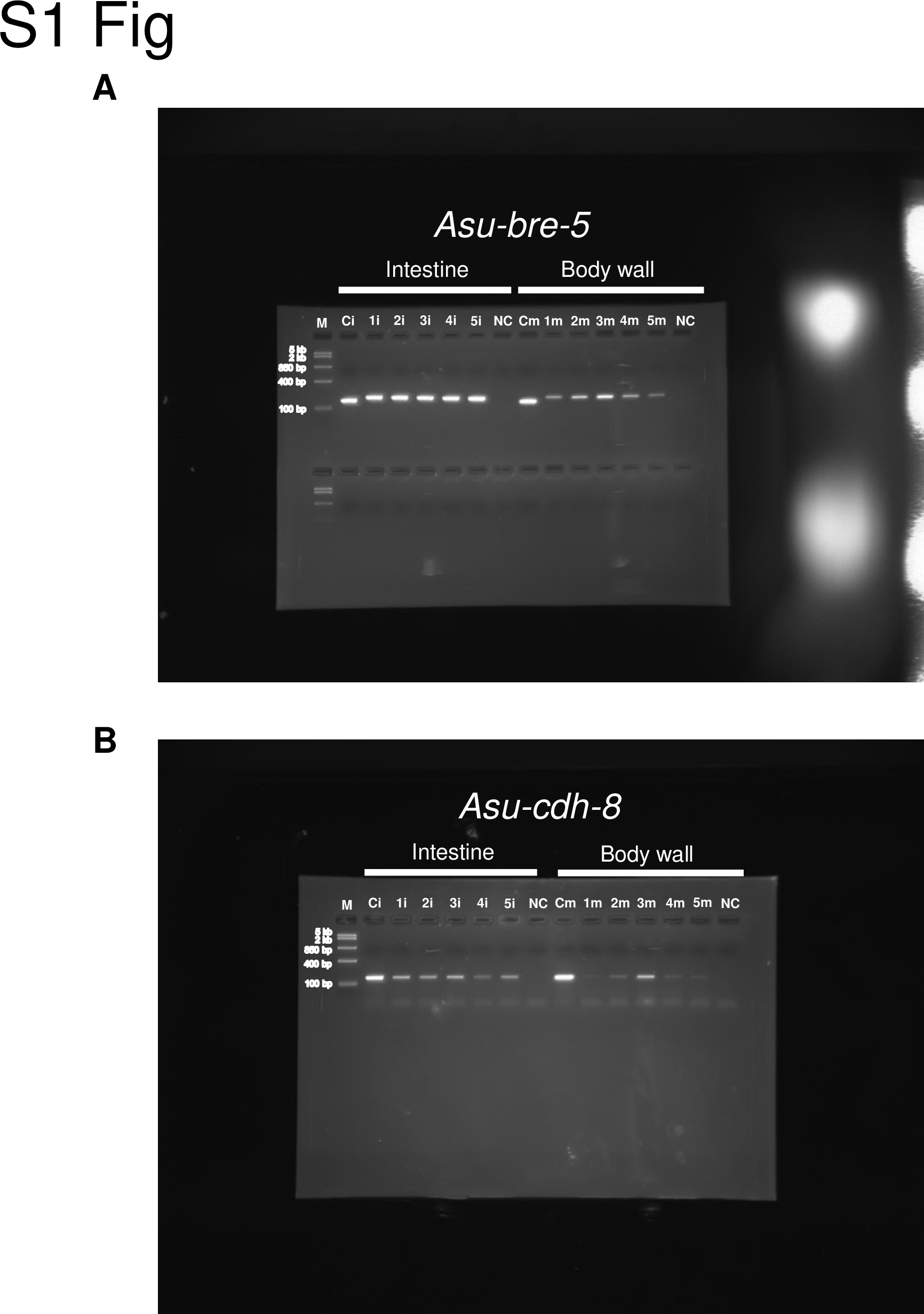

Supplement: S1 Fig — Original uncropped gel pictures from Fig 2 showing RT-PCR analysis of intestine (1i, 2i, 3i, 4i, 5i) and muscle bag (1b, 2b, 3b, 4b, 5b) of five separate female A. suum worms. Each lane represents the intestine or muscle bag of an individual worm. Asu-gapdh from the intestine (Ci) or muscle bag (Cb) was used as a positive control. N.C. = negative control, no cDNA template present. M = FastRuler Middle Range DNA Ladder (ThermoFisher Scientific). A) Asu-bre-5, B) Asu-cdh-8 Images were taken under UV light with an exposure setting of 3 seconds per 1 frame. (TIF) [file ppat.1011835.s001.tif]

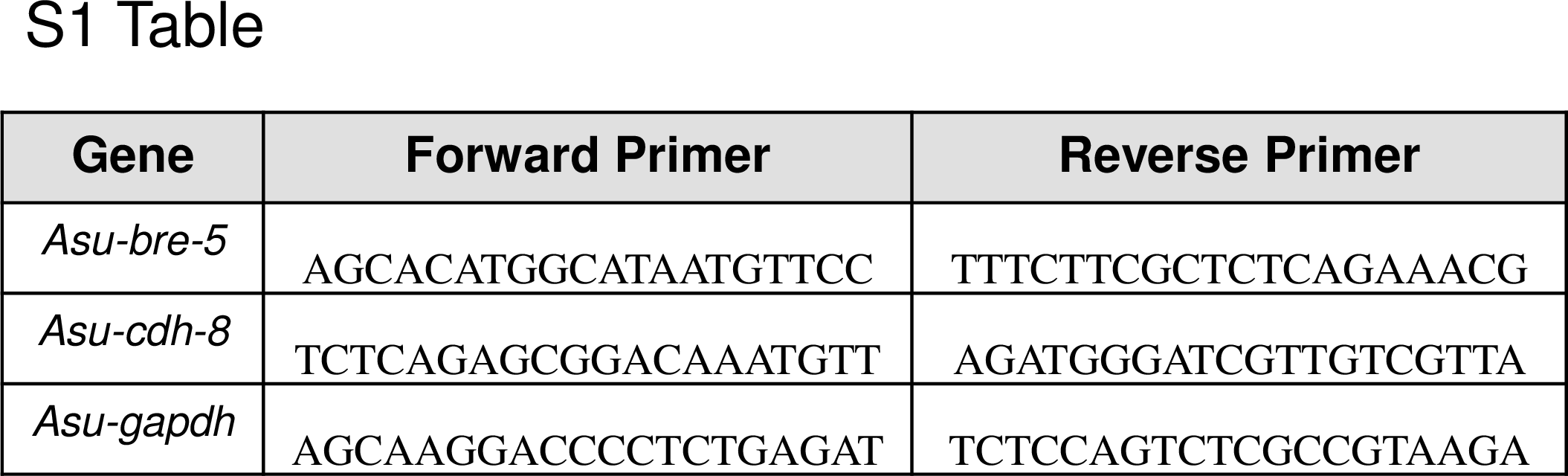

Supplement: S1 Table — (TIF) [file ppat.1011835.s002.tif]

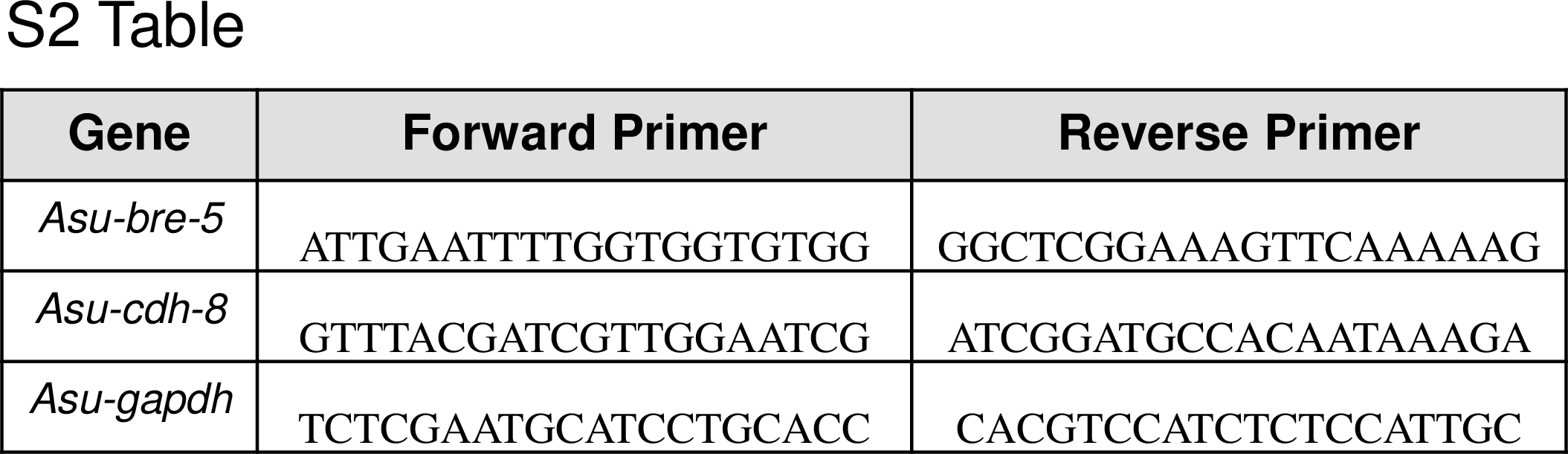

Supplement: S2 Table — (TIF) [file ppat.1011835.s003.tif]

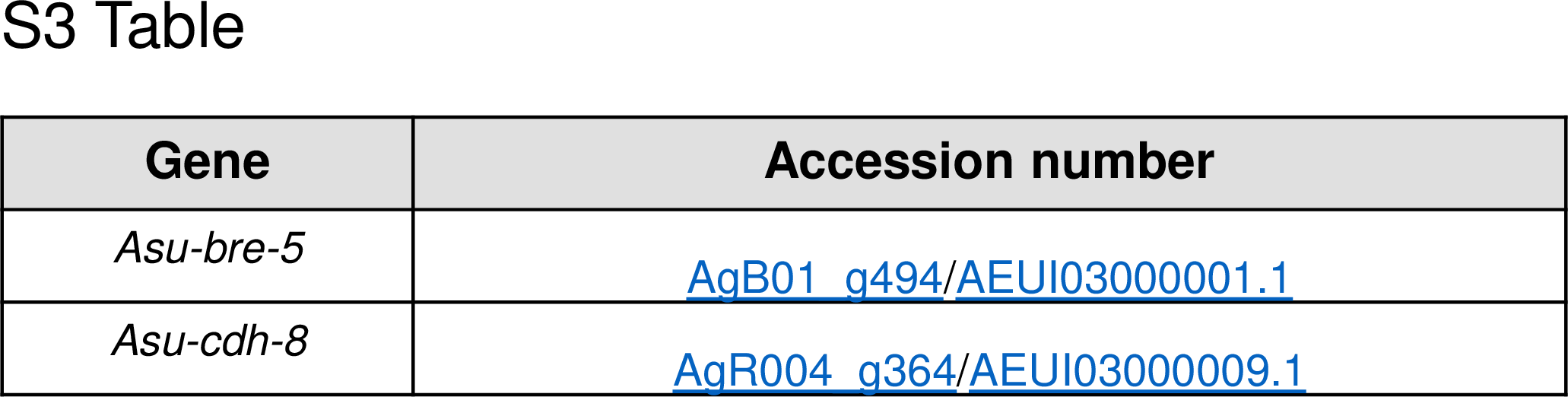

Supplement: S3 Table — (TIF) [file ppat.1011835.s004.tif]
